# Differential proteomic analysis of fetal and geriatric lumbar nucleus pulposus: immunoinflammation and age-related intervertebral disc degeneration

**DOI:** 10.1186/s12891-020-03329-8

**Published:** 2020-06-02

**Authors:** Chensheng Qiu, Xiaolin Wu, Jiang Bian, Xuexiao Ma, Guoqing Zhang, Zhu Guo, Yan Wang, Yandong Ci, Qizun Wang, Hongfei Xiang, Bohua Chen

**Affiliations:** 1 Medical College of Qingdao University, Qingdao, 266000 China; 2 Department of Orthopedic Surgery, Affiliated Hospital of Qingdao University, Qingdao, 266000 China; 3 Department of Orthopedic Surgery, Qingdao Municipal Hospital (Group), Qingdao, 266011 China; 4 Qingdao Eye Hospital of Shandong First Medical University, Qingdao, 266000 China; 5 The Eighth People’s Hospital of Qingdao, Qingdao, 266000 China

**Keywords:** Intervertebral disc degeneration, Proteomics, Tandem mass tag, Aging, Inflammatory response

## Abstract

**Background:**

Intervertebral disc degeneration (IVDD) is a major cause of low back pain. Although the mechanism of degeneration remains unclear, aging has been recognized as a key risk factor for IVDD. Most studies seeking to identify IVDD-associated molecular alterations in the context of human age-related IVDD have focused only on a limited number of proteins. Differential proteomic analysis is an ideal method for comprehensively screening altered protein profiles and identifying the potential pathways related to pathological processes such as disc degeneration.

**Methods:**

In this study, tandem mass tag (TMT) labeling was combined with liquid chromatography-tandem mass spectrometry (LC-MS/MS) for differential proteomic analysis of human fetal and geriatric lumbar disc nucleus pulposus (NP) tissue. Parallel reaction monitoring (PRM) and Western blotting (WB) techniques were used to identify target proteins. Bioinformatic analyses, including Gene Ontology (GO) annotation, domain annotation, pathway annotation, subcellular localization and functional enrichment analyses, were used to interpret the potential significance of the protein alterations in the mechanism of IVDD. Student’s t-tests and two-tailed Fisher’s exact tests were used for statistical analysis.

**Results:**

Six hundred forty five proteins were significantly upregulated and 748 proteins were downregulated in the geriatric group compared with the fetal group. Twelve proteins were verified to have significant differences in abundance between geriatric and fetal NP tissue; most of these have not been previously identified as being associated with human IVDD. The potential significance of the differentially expressed proteins in age-related IVDD was analyzed from multiple perspectives, especially with regard to the association of the immunoinflammatory response with IVDD.

**Conclusions:**

Differential proteomic analysis was used as a comprehensive strategy for elucidating the protein alterations associated with age-related IVDD. The findings of this study will aid in the screening of new biomarkers and molecular targets for the diagnosis and therapy of IVDD. The results may also significantly enhance our understanding of the pathophysiological process and mechanism of age-related IVDD.

## Background

Low back pain (LBP) severely affects human health in the modern world, placing enormous burdens on patients and society [1]; unfortunately, the pathogenesis of LBP is not entirely understood. Intervertebral disc degeneration (IVDD) is a well-known cause of LBP, especially in elderly people [2, 3]. The pathogenesis of IVDD is complex and diverse, with aging considered to be the most significant risk factor [4, 5]. Thus, it is critical to understand the pathophysiological changes associated with disc aging in order to develop an effective treatment for age-related IVDD.

IVDD begins in the nucleus pulposus (NP), the core component of the disc [6]. The anatomic and pathophysiological characteristics of NP tissue change rapidly after birth, causing earlier age-related degeneration in intervertebral discs than other tissues [7–13]. It has been reported that IVDD begins at the age of approximately 15 ~ 20 years, but some recent studies have demonstrated that it may actually begin much earlier, tracing back as early as the infancy stage [10]. Organismal aging and its ensuing pathophysiological changes can be reflected at the protein level. However, previous research on age-related IVDD has focused on a limited number of proteins and pathways. Animal models and human body fluids are generally used to explore the mechanism of IVDD but may not directly reflect the pathophysiological changes that occur in discs. Overall, few studies have evaluated the biological characteristics of intervertebral discs through comprehensive protein profiling, especially in human NP. Proteomics is a discipline that dynamically studies the composition, function and relation of all proteins under specific physiological or pathological conditions from a holistic perspective [14]. Differential proteomic analysis, which focuses on screening and identifying changes by comprehensive protein profiles between different samples, is an ideal approach for assessment of protein alterations. As proteomic technologies have continued to improve, stable isotope labeling, especially tandem mass tag (TMT) labeling, combined with mass spectrometry (MS), has become an important method for protein quantification [15]. Therefore, comprehensive analysis of protein alterations between fetal and geriatric NP via differential proteomic strategy will provide meaningful information that may be helpful in understanding the mechanism of age-related IVDD.

In this study, the differentially expressed proteins between fetal and geriatric lumbar disc NP tissues were screened and analyzed by TMT labeling combined with liquid chromatography (LC)-tandem MS (MS/MS). Parallel reaction monitoring (PRM) [16] and Western blotting (WB) techniques were employed to identify target proteins that may be closely related to age-related IVDD. Additionally, bioinformatic analyses, including Gene Ontology (GO) annotation, subcellular localization, domain annotation, pathway annotation, and functional enrichment analyses, were used to interpret the potential significance of the altered protein profiles related to mechanisms of IVDD.

## Methods

### Participants and sample collection

Human fetal lumbar L4/5 intervertebral disc NP samples were collected to form the fetal group (*n* = 5). The specimens were derived from fetuses whose mothers had to terminate their pregnancies due to severe disease or trauma. The fetuses were delivered to the laboratory within 4 h after induced labor. The gestational ages ranged from 31 to 35 weeks (average 33.2 ± 1.6 weeks), which is very close to full term, and the fetuses comprised 2 males and 3 females. L4/5 intervertebral disc NP samples from geriatric patients were collected during spinal fusion for chronic LBP to form the geriatric group (*n* = 5). Magnetic resonance imaging confirmed that the L4/5 intervertebral discs were degenerated but did not exhibit apparent protrusions or sequestrations. The degree of degeneration was Grade IV, as classified by the Pfirrmann grading system [17]. Patients with lumbar disc herniation, lumbar trauma history, spinal tumors, spinal infection, smoking history, diabetes, obesity, and autoimmune diseases were excluded. The geriatric patients ranged in age from 56 to 71 years (average 63.2 ± 6.2 years) and comprised 3 males and 2 females. The clinical diagnosis was performed by two spine surgeons and a radiologist based on imaging and physical examination and was confirmed during surgery. After separation of the NP using a stereomicroscope, blood and impurities on the tissue surface were thoroughly removed by washing with sterile normal saline; the samples were then stored in liquid nitrogen.

### Protein extraction

Each sample was ground to a powder in liquid nitrogen. Four volumes of lysis buffer (2 mM EDTA [Sigma, USA], 8 M urea [Sigma], and 1% Protease Inhibitor Cocktail [Calbiochem, Germany]) were added. The supernatant was collected after ultrasonic processing and centrifugation. A BCA kit was used to determine the protein concentration (Beyotime, China).

### Trypsin digestion

5 mM dithiothreitol (Sigma) was used to reduce the protein solution at 56 °C. 30 min later, at room temperature, 11 mM iodoacetamide (Sigma) was added to alkylate the solution for 15 min in the dark. Triethylammonium bicarbonate (TEAB) (100 mM) (Sigma) was used to dilute the urea concentration of the protein sample to less than 2 M. For the first digestion overnight, trypsin (Promega, USA) was added to the sample (mass ratio, 1:50 trypsin:protein). For the next 4 h, a second digestion was performed (mass ratio, 1:100 trypsin:protein).

### TMT labeling

The peptides were desalted with a Strata X C18 SPE column (Phenomenex, USA) and vacuum freeze-dried after digestion. After being dissolved in 0.5 M TEAB, the peptides were labeled with a TMT kit (Thermo Fisher Scientific, USA) according to the manufacturer’s protocol. Peptides derived from fetal NP samples were labeled with TMT tags of 126, 127 N, 127C, 128 N and 128C, and geriatric NP samples were labeled with TMT tags of 129 N, 129C, 130 N, 130C and 131. The labeled peptides were mixed, desalted and dried.

### High performance liquid chromatography (HPLC) fractionation

The peptide mixtures were fractionated by high-pH reverse-phase HPLC using an Agilent 300Extend-C18 column. In brief, the peptides were first divided into 60 fractions with a linear gradient of acetonitrile (8 to 32%, pH 9.0) over 1 h. Then the peptides were combined into 18 fractions and vacuum dried. The detailed fraction sequence is shown in Additional file 1 (HPLC Fraction Sequence).

**Fig. 1:**
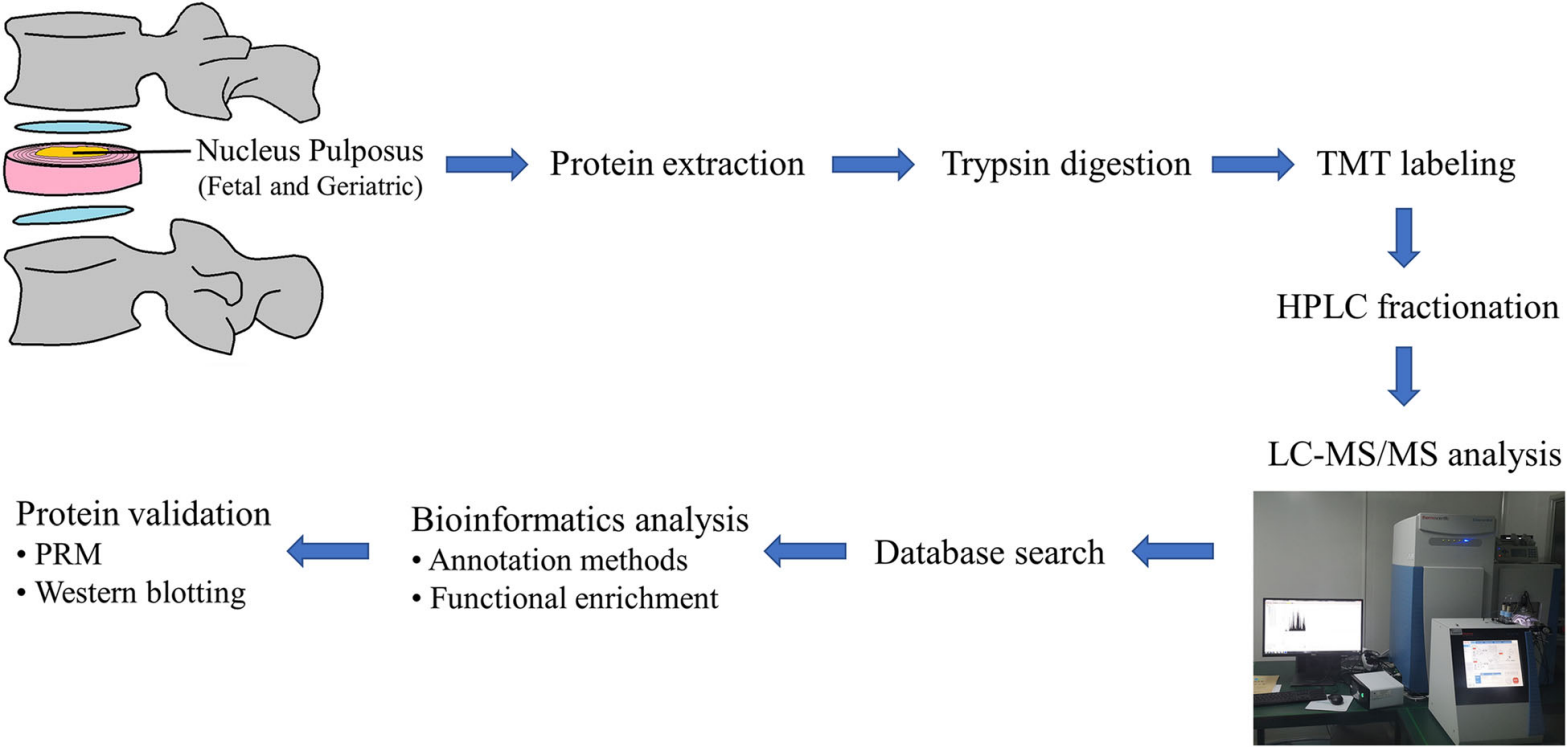
Workflow for the proteomic analysis. Fetal and geriatric NP samples were collected for analysis using TMT combined with LC-MS/MS. After proteins were identified, annotation and functional enrichment methods were used for bioinformatic analysis. PRM and WB were used to validate crucial differentially expressed proteins

### LC-MS/MS analysis

Tryptic peptides were dissolved in solvent A (2% acetonitrile and 0.1% formic acid) and separated via a gradient of solvent B (90% acetonitrile and 0.1% formic acid) as follows: 9 to 25% (26 min), 25 to 38% (8 min), increasing to 80% (3 min) and holding at 80% (3 min). Separation was performed in an EASY-nLC 1000 ultra-performance LC (UPLC) system (Thermo Fisher Scientific). The flow rate was 700 nL/min. After injection into a nanospray ionization (NSI) source, the peptides were analyzed by MS/MS using an Orbitrap Fusion (Thermo Fisher Scientific). The detailed parameter settings are shown in Additional file 1 (LC-MS/MS Analysis Parameters).

### Database search

The raw MS/MS data were processed by MaxQuant search engine (v.1.5.2.8) against the SwissProt human database (20,317 sequences). A contaminant database and a reverse decoy database were combined. The detailed parameter settings are shown in Additional file 1 (Database Search Parameters).

### Bioinformatic methods

GO annotation, domain annotation, pathway annotation and subcellular location analyses were performed. The UniProt-GOA database was used for investigating the GO-annotated proteome, via InterProScan software. The proteins were classified by their GO annotations into three categories: biological process, cellular component, and molecular function. The InterPro database was used to annotate the domains of the identified proteins. The Kyoto Encyclopedia of Genes and Genomes (KEGG) database was employed for protein pathway annotation. WoLF PSORT [18] was used to analyze the subcellular localization. The altered proteins were subjected to functional enrichment (GO, domain, and KEGG pathway) analyses. A two-tailed Fisher’s exact test was used for enrichment analysis. A corrected *p*-value of < 0.05 was considered to indicate significance. The URLs of the databases and software programs noted above are shown in Additional file 1 (Software and Database URLs).

### Protein validation by PRM

The identified proteins were ranked in terms of their degree of change and classified based on their known functions individually. Target proteins were screened for further verification via PRM, a highly targeted MS method, based on their traits and the pathophysiological mechanisms of IVDD. In brief, after being dissolved in solvent A, the tryptic peptides were separated through a gradient of solvent B as follows: 7 to 25% (40 min), 25 to 35% (12 min), increasing to 80% (4 min) and holding at 80% (4 min). Separation was performed in the EASY-nLC 1000 UPLC system. The constant flow rate was 350 nL/min. The peptides were subjected to NSI source, followed by MS/MS in a Q Exactive™ Plus (Thermo Fisher Scientific) coupled online to the UPLC. Each sample was measured separately. The raw data were searched via Skyline (v.3.6) [19]. The detailed parameter settings are shown in Additional file 1 (PRM Analysis Parameters and Skyline Parameters).

### Protein validation by WB

Analysis of the results indicated that the abundance of LTA (protein accession number: P01374) was significantly increased in the geriatric group. We carried out WB to detect the expression of LTA for further verification. In brief, 25 μg of protein from each sample was separated by 12% SDS-PAGE. After transferring the proteins to PVDF membranes (Beyotime), the membranes were subjected to immunoblotting with anti-LTA antibodies (1:1000 dilution, Affinity, USA) at 4 °C overnight. They were then incubated with HRP-conjugated secondary antibodies (1:5000 dilution, Proteintech, China) after washing with PBST buffer. Protein expression was detected by enhanced chemiluminescence.

### Statistical analysis

Statistical analysis was performed using SPSS Statistics 22.0. The average value of each identified protein with each group was calculated. Student’s t-tests and two-tailed Fisher’s exact tests were used to evaluate the significance of differences between the two groups. Only differences with a corrected *p*-value of < 0.05 and a fold change of > 1.50 or < 0.67 (1/1.50) were considered to indicate differential abundance.

## Results

### Overview of differentially expressed proteins

The workflow for the proteomic analysis is shown in Fig. 1. The mass error distribution and peptide lengths were used to assess each identified peptide. The prepared samples met the requirements (Additional file 2). Coefficients of variation (CV) [20] was used to evaluate the stability of the data upon examining the variations in abundance among individuals in the same sample group. In the fetal group, 99.3% of the proteins had CVs of less than 30, and 92.8% had CVs of less than 15%. In the geriatric group, 96.4% of the proteins had CVs of less than 30, and 77.4% had CVs of less than 15%. These findings indicated that the differences among the samples in each group were small and that the data were relatively stable (Additional file 3).

In this study, 3259 proteins were identified, among which 2691 were quantified. A quantitative geriatric/fetal ratio of > 1.50 indicated upregulation; a ratio of < 0.67 (1/1.50) indicated downregulation. Six hundred forty five proteins were upregulated, while 748 were downregulated. The MS information is shown in Additional file 3.

### Functional classification of differentially expressed proteins

#### GO functional classification

GO analysis links information between genes and gene products, such as proteins. In this study, the quantified differentially expressed proteins were examined based on their biological properties as identified by GO analysis. The upregulated and downregulated GO annotation classifications are shown in Additional file 4.

#### Subcellular localization classification

**Fig. 2:**
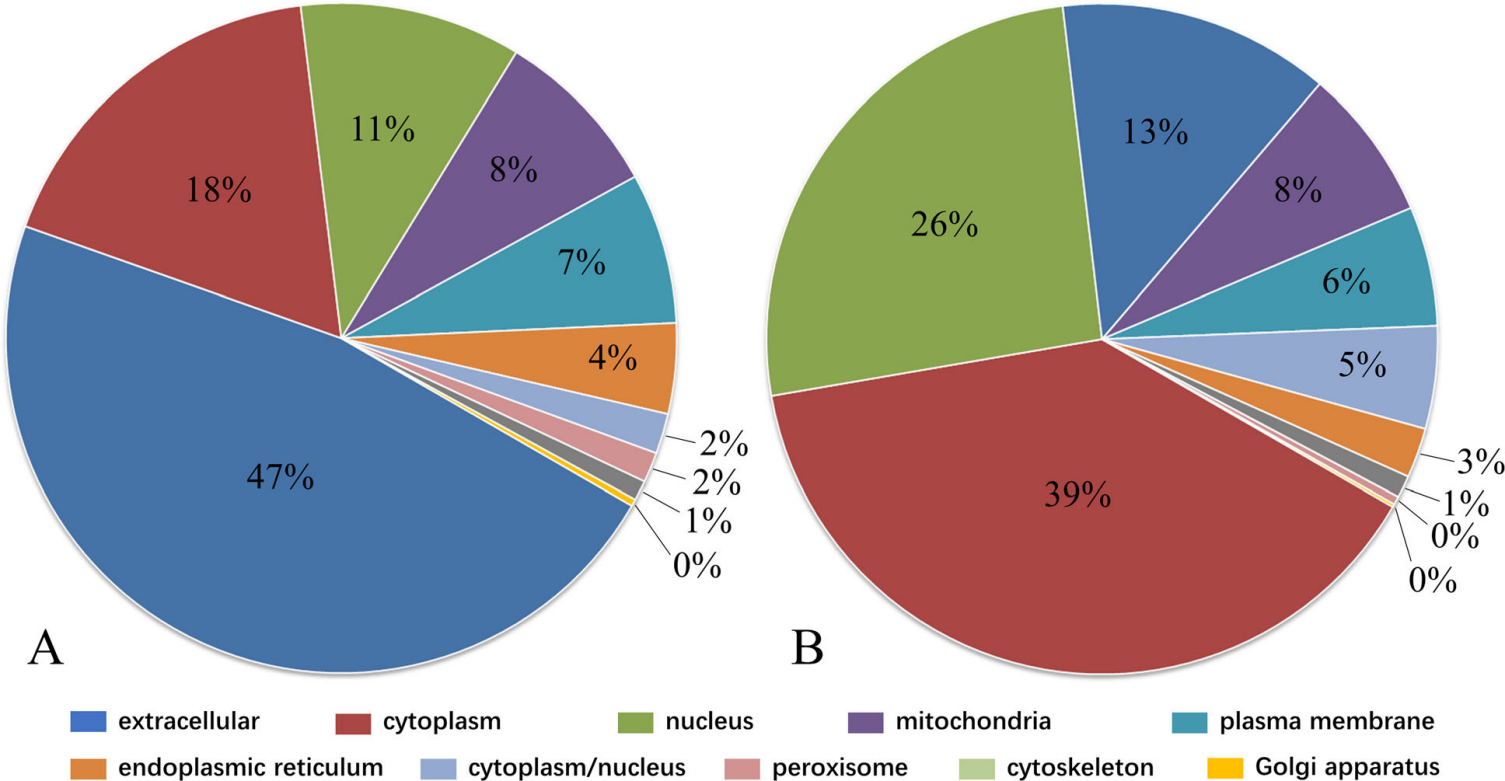
Subcellular localization classification. **a** Proportions of upregulated proteins in various subcellular locations (geriatric/fetal). **b** Proportions of downregulated proteins in various subcellular locations (geriatric/fetal)

The subcellular localizations of differentially expressed proteins were predicted and classified by WoLF PSORT software (Fig. 2). The upregulated proteins were concentrated mostly in the extracellular space (291, 47%), followed by the cytoplasm (109, 18%) and nucleus (66, 11%); the downregulated proteins were concentrated mostly in the cytoplasm (291, 39%), followed by the nucleus (193, 26%) and extracellular space (98, 13%) (geriatric/fetal). The proportions of upregulated and downregulated proteins in the mitochondria, plasma membrane, endoplasmic reticulum, cytoplasm/nucleus, peroxisome, cytoskeleton and Golgi apparatus regions were similar.

#### Enrichment analysis

Enrichment analysis was performed to detect the functional trends of the altered proteins based on their annotations. The *p*-values obtained in the enrichment test were converted to negative logarithm (−log10) values. A larger converted value indicates a more significant enrichment of that functional type.

#### GO annotation enrichment analysis

**Fig. 3:**
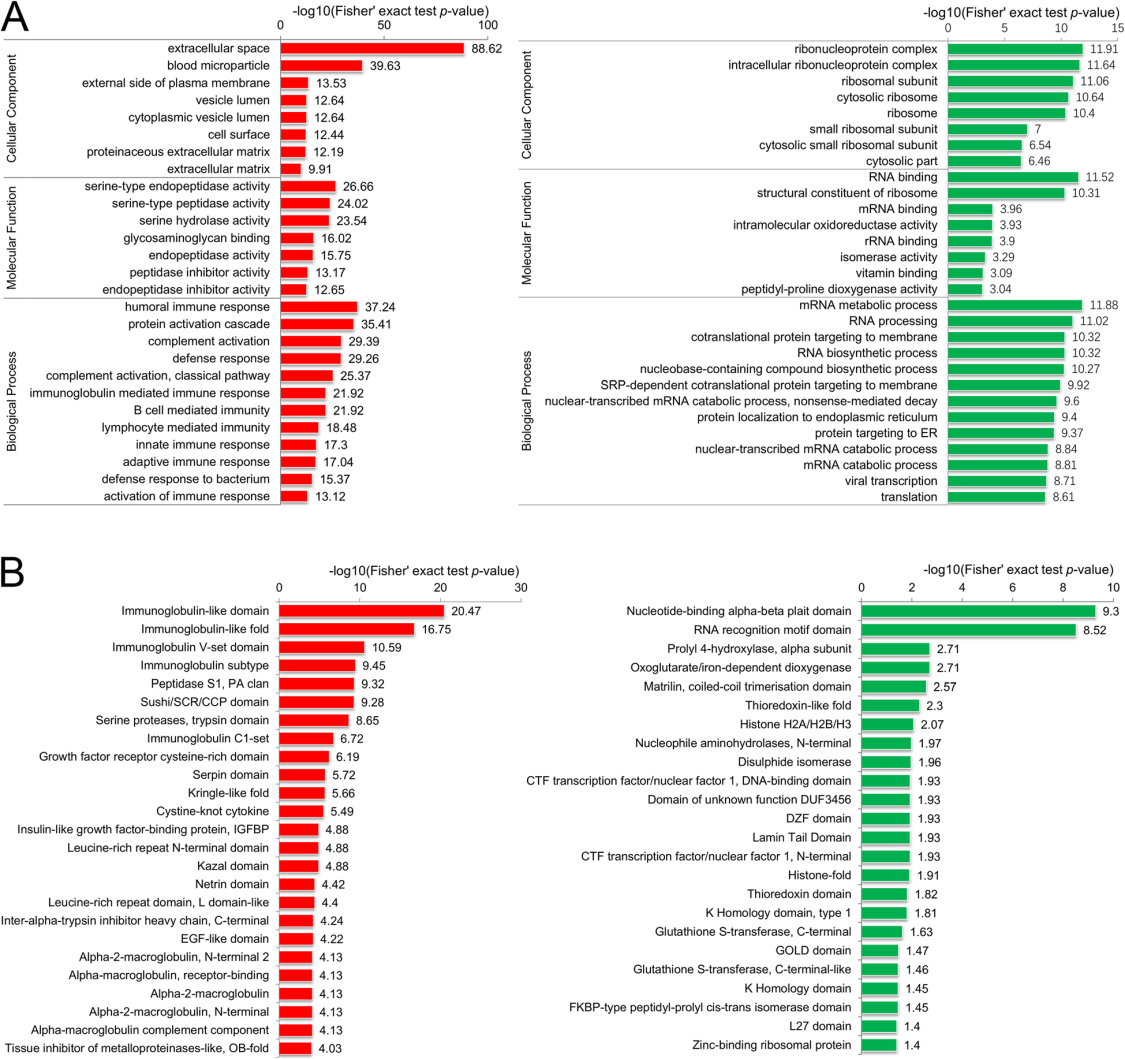
GO annotation and domain annotation enrichment analysis results (geriatric/fetal). **a** GO annotation enrichment analysis results. The red bars show the degrees of significance of the upregulated proteins, and the green bars show the degrees of significance of the downregulated proteins. **b** Domain annotation enrichment analysis results. The red bars show the degrees of significance of the upregulated protein domains, and the green bars show the degrees of significance of the downregulated protein domains

GO annotation enrichment analysis was performed on the subitems in the three level-1 categories. The main results of the GO annotation enrichment analysis are shown in Fig. 3a. In particular, the upregulated proteins (geriatric/fetal) were enriched mainly for terms related to serine-related activity, various types of immune response, complement activation, and defense response; the downregulated proteins were enriched mainly for terms related to RNA biosynthetic process, RNA binding and structural components of the ribosome. These results reflect the activation of immunoinflammatory responses and the decreases in protein synthesis capacity in degenerated discs.

#### Domain annotation enrichment analysis

Protein domains are local, compact structural units that cannot be further subdivided and are considered “evolutionary units” of protein sequences. The domain enrichment analysis results are shown in Fig. 3b. In particular, the upregulated proteins (geriatric/fetal) were enriched mainly for immunoglobulin-related and serine protease-related domains. These results reflect the activation of the inflammatory response in degenerated discs from a more microscopic perspective.

#### KEGG pathway enrichment analysis

**Fig. 4:**
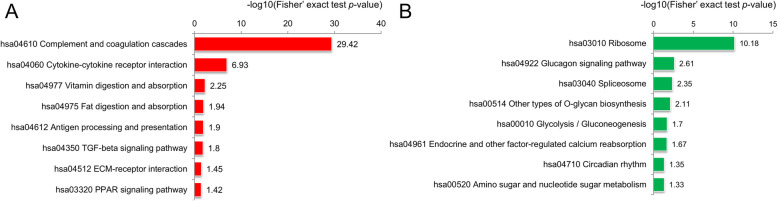
KEGG pathway enrichment analysis results (geriatric/fetal). **a** The red bars represent the degrees of significance of upregulated protein enrichment. **b** The green bars represent the degree of significance of downregulated protein enrichment

**Fig. 5:**
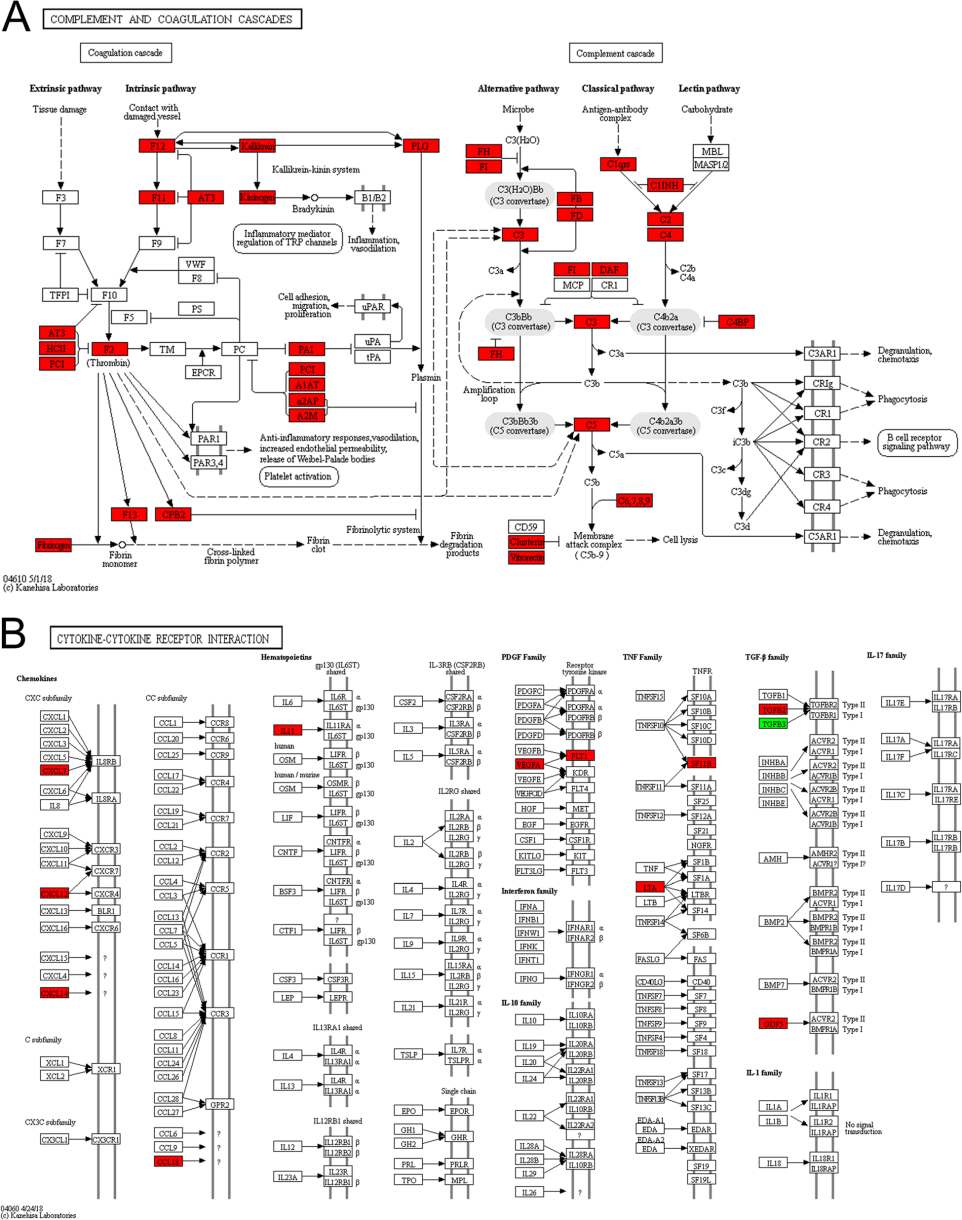
Complement and coagulation cascade pathways (hsa04610) and the cytokine-cytokine receptor interaction pathway (hsa04060) (geriatric/ fetal). **a** In the coagulation cascade pathway, coagulation factors, including FII, FXI, FXII, and FXIII, and anticoagulation factors, including AT3, HCII, and PCI, were significantly upregulated. In particular, the intrinsic coagulation pathway was activated. Regarding the complement cascade pathway, C1-C9, FB, FH, FD, and FI were significantly upregulated. Both the classic and alternative pathways of the complement cascade were activated. **b** In the cytokine-cytokine receptor interaction pathway, the following proteins were significantly upregulated: CXCL7, CXCL12, CXCL14, and CCL18 in the chemokine family; IL-11 in the hematopoietin family; VEGFA in the PDGF family; and LTA in the TNF family. TGFB2 and GDF5 in the TGF-β family were also upregulated, whereas TGFB3 was downregulated

The KEGG is an information network of known molecular interactions that includes information on metabolism, genes, cellular processes, and diseases. The results showed that the upregulated proteins (geriatric/fetal) were enriched primarily in the complement and coagulation cascades pathway (hsa04610) and the cytokine-cytokine receptor interaction pathway (hsa04060). The downregulated proteins were enriched mainly in the ribosome pathway (hsa03010) (Fig. 4). In the figure, red is used for KEGG pathways enriched for upregulated proteins, while green is used for KEGG pathways enriched for downregulated proteins. Among these pathways, complement and coagulation cascades (hsa04610) and cytokine-cytokine receptor interaction (hsa04060) are present, as shown in Fig. 5.

Ribosomes are the main sites of intracellular protein synthesis and are particularly abundant in rapidly proliferating cells. The downregulated proteins (geriatric/fetal) were enriched mainly in the ribosome pathway (hsa03010) (Fig. 4b). In addition, the quantities and proportions of proteins that localized to the cytoplasm and nucleus, involved in the RNA biosynthetic process, and involved in the RNA binding process were reduced (Fig. 2 and Fig. 3a) in degenerated discs. These findings reflect the decreases in protein synthesis ability in aged and degenerated intervertebral discs.

#### Protein validation by PRM

Eleven target proteins in human NP tissue that had not previously been clearly reported to be involved in IVDD were screened based on well-known theories and findings related to the pathophysiology of IVDD. PRM was used to further validate the protein alterations, and the main results are provided in Table 1. The fragment ion peak maps are shown in Additional file 5.

**Table 1:** Protein Quantification by PRM (Geriatric/Fetal)

Protein Accession	Protein Name	Gene Name	Ratio (PRM)	*p*-value (PRM)	Ratio (TMT)
P20809	Interleukin-11	IL11	+infinite	7.98E-11	31.05
P11142	Heat shock cognate 71 kDa protein	HSPA8	0.17	5.30E-06	0.75
O15232	Matrilin-3	MATN3	0.01	5.01E-09	0.38
P10909	Clusterin	CLU	35.76	4.08E-08	16.73
Q08431	Lactadherin	MFGE8	1.27	1.60E-02	2.14
P98066	Tumor necrosis factor-inducible gene 6 protein	TNFAIP6	78.01	9.57E-12	14.13
Q9NR99	Matrix-remodeling-associated protein 5	MXRA5	12.29	7.39E-11	5.88
Q16610	Extracellular matrix protein 1	ECM1	4.08	8.51E-12	5.53
P05121	Plasminogen activator inhibitor 1	SERPINE1	176.51	1.85E-07	12.06
Q9NRA1	Platelet-derived growth factor C	PDGFC	23.61	2.95E-09	22.42
P50502	Hsc70-interacting protein	ST13	0.14	8.67E-08	0.54

#### Protein validation by WB

**Fig. 6:**
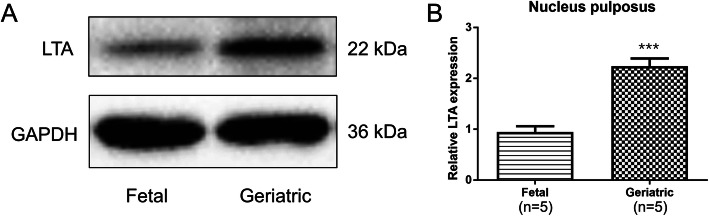
Protein validation by WB. **a** Protein lysates from the NP samples of fetuses and geriatric patients were probed with anti-LTA (22 kDa) and anti-GAPDH (36 kDa) antibodies. The level of LTA in the geriatric group was significantly higher than those in the fetal group. **b** Results of quantitative analysis of LTA protein levels shown in “A”. All data are displayed as the mean ± SD, *** *p* < 0.001

After conducting PRM, we identified another protein, LTA, that may be closely related to IVDD based on the results of TMT labeling. Compared with the fetal group, the geriatric group exhibited significantly higher expression of LTA (ratio: 32.74, *p*-value: 3.07E-06). Therefore, we performed WB for secondary verification. The WB results confirmed that the expression of LTA was significantly increased in the geriatric group, consistent with the quantitative proteomic analysis results (Fig. 6).

## Discussion

IVDD is a complex clinical condition induced by numerous factors that can eventually lead to the occurrence and development of degenerative disc disorders. In this study, quantitative differential proteomic analysis was used to systematically screen differentially abundant proteins between human fetal and geriatric lumbar disc NP tissues. Through bioinformatics techniques, the relationships of the differentially expressed proteins to age-related IVDD, especially regarding immunoinflammatory responses, were examined. We also identified 12 differentially expressed proteins closely related to age-related IVDD in human intervertebral discs, LTA, IL11, HSPA8, MATN3, CLU, MFGE8, TNFAIP6, MXRA5, ECM1, SERPINE1, PDGFC and ST13, most of which are reported here for the first time.

The immunoinflammatory response plays a critical role in the pathogenic process of age-related IVDD. Activation of autoimmunity and abnormal expression of inflammatory cytokines lead to disc cell death and dysfunction, increased degradation of the extracellular matrix (ECM) and decreased protein synthesis, thus accelerating IVDD [21]. The enrichment analysis in this study revealed that the upregulated proteins (geriatric/fetal) were closely associated with various types of immune responses, complement activation, and serine protease-related activity. Many cytokines in the cytokine-cytokine receptor interaction pathway that are involved in the inflammatory response were significantly upregulated, indicating that immunoinflammatory responses were activated in aged and degenerated intervertebral discs. Among these upregulated cytokines, LTA and IL11 particularly attracted our attention. The proteomic alterations were further verified by PRM and WB and the potential molecular mechanism related to these findings is currently under investigation by our research group. The present results suggest that the cytokines LTA and IL11 are closely related to the process of IVDD. The cytokine CXCL12 was shown to be upregulated in the degenerated discs in this study, which is consistent with previous research findings and may have potential value in research on intervertebral disc repair [22]. The activated immunoinflammatory response and the changes in cytokines may be related to phenotypic changes in senescent NP cells and autoimmunity; however, their causality in the pathogenesis of age-related IVDD remains unknown.

IVDD is an age-related degenerative disease, and the degree of IVDD gradually increases with age [5]. The number of senescent NP cells in an intervertebral disc is significantly and positively correlated with the degree of degeneration [4]. Disc cells undergo phenotypic changes during aging and senescence. The proinflammatory phenotype of senescent cells is defined as a senescence-associated secretory phenotype (SASP). Although the overall protein synthesis ability is decreased in senescent cells, inflammatory cytokines are abnormally overexpressed. Previous studies have demonstrated that these inflammatory cytokines play important roles in deteriorating the disc microenvironment and eventually accelerate IVDD, thus creating a vicious cycle [23].

Intervertebral discs are immunologically privileged tissues [7]. After birth, the NP is isolated from the blood circulation and becomes the largest avascular area; therefore, the NP is immune-privileged throughout life. This unique anatomical feature renders NP tissue autoantigenic. NP has been suggested to trigger an autoimmune response if the immune-privileged state of the disc is destroyed and the NP is exposed to the immune system [7]; in such case, immune cells and inflammatory cytokines subsequently induce NP cell death and ECM degradation, which may cause or accelerate IVDD. Notably, notochord-secreted molecules restrict axons and/or blood vessels from growing into improper areas [24]; however, this ability gradually diminishes with disc aging. Studies have shown that vascularized granulation tissue can extend from the outer annulus fibrosus into the NP in degenerated discs [25, 26]. Our study also supports this concept. For example, the intrinsic coagulation pathway, which is usually activated due to damage to vessel walls, was significantly activated in degenerated NP tissue. Additionally, vascular endothelial growth factor A (VEGFA), which plays important roles in angiogenesis, vasculogenesis and endothelial cell growth, was upregulated (geriatric /fetal). Given our results and those of previous studies [24–26], we speculate that new blood vessels can grow into deep NP tissue during disc aging and degeneration and that the destruction and reconstruction of blood vessels can lead the antigenic components in NP tissue to be exposed to the circulatory system. As a result, the antigenic components from damaged NP tissue stimulate immunoinflammatory responses when interacting with immune cells, ultimately leading to early IVDD. The alterations of several cytokines in peripheral blood of patients with degenerative disc diseases are consistent with those found in intervertebral discs, such as interleukin-6 and CCL5 [21, 27, 28]. Vascularization of degenerative discs may be a cause of the local inflammatory reaction, which is reflected in the blood circulation system. Therefore, new blood biomarkers found through further studies on the relationship between cytokines in peripheral blood and discs will have potential significance on the early diagnosis and monitoring of IVDD progression. Taken together, the proteomic results from this study suggest a possible pathological condition in which aging and degeneration of the disc may result in vascular growth into the deeper disc.

Ten additional altered proteins were identified: HSPA8, MATN3, CLU, MFGE8, TNFAIP6, MXRA5, ECM1, SERPINE1, PDGFC and ST13. Most of these proteins have not been previously identified to be associated with IVDD in human. These proteins are involved in cell proliferation, cell migration, inflammatory responses, vascular proliferation, fibrosis, ECM degradation, autophagy, apoptosis, cell senescence, tissue remodeling, and other biological processes closely related to IVDD [29–37]. The potential molecular mechanism of the involvement of HSAP8 and MATN3 in IVDD has been investigated by our research group, and some of the research findings have already been published [38].

The lack of an absolutely healthy disc source is the most important restrictive factor in basic research on IVDD. Relatively healthy scoliotic/traumatic disc specimens obtained from young patients have often been selected as normal control specimens in previous studies [39, 40]. However, an increasing number of studies have shown that even these disc specimens have pathological changes, with some exhibiting early degeneration [41, 42]. Other studies have shown that discs in full-term or near-full-term fetuses are very similar to healthy discs in young people and may be selected as ideal healthy control specimens [43, 44]. Nevertheless, the influences of disc growth and developmental factors on experimental results should not be ignored. As mentioned earlier, recent research progress has demonstrated that degeneration of intervertebral discs may actually occur much earlier than previously reported, tracing back as early as the infancy stage [10]. Therefore, even with the limitations above, our differential proteomic analysis between human fetal and geriatric NP tissues still has far-reaching implications for understanding disc aging and degeneration. Despite the small sample size and individual variance in this study, the potential biological targets identified are clinically informative especially because these specimens were from humans. Further investigations will use the database generated by this study to explore the potential mechanisms of age-related IVDD and their significance.

## Conclusion

In conclusion, our differential proteomic analysis comprehensively profiled protein alterations that are potentially associated with age-related IVDD. The results provide a useful database for further investigations into the pathophysiological processes and molecular biological mechanisms of age-related IVDD, especially regarding the association of the immunoinflammatory response with IVDD. New potential biomarkers and molecular targets for the diagnosis and therapy of IVDD have been identified.

## Supplementary information

**Additional file 1.** Parameter Settings. Detailed parameter settings for the HPLC fraction sequence, LC-MS/MS analysis, database search, PRM analysis, Skyline analysis and database URLs.

**Additional file 2.** Quality control plots. The mass error and length distributions of peptides.

**Additional file 3.** Information identified by MS.

**Additional file 4.** GO annotation classification.

**Additional file 5.** PRM fragment ion peak maps.

## Data Availability

The mass spectrometry proteomics data have been deposited to the ProteomeXchange Consortium (http://proteomecentral.proteomexchange.org) via the iProX partner repository [45] with the dataset identifier PXD015000.

## Abbreviations

IVDD: Intervertebral disc degeneration
TMT: Tandem mass tag
LC: Liquid chromatography
LBP: Low back pain
NP: Nucleus pulposus
ECM: Extracellular matrix
PRM: Parallel reaction monitoring
WB: Western blotting
GO: Gene Ontology
TEAB: Triethylammonium bicarbonate
HPLC: High performance liquid chromatography
UPLC: Ultra-performance liquid chromatography
MS/MS: Tandem mass spectrometry
KEGG: Kyoto Encyclopedia of Genes and Genomes
CV: Coefficients of variation
VEGFA: Vascular endothelial growth factor A
SASP: Senescence-associated secretory phenotype
MS: Mass spectrometry
NSI: Nanospray ionization
