# Supplementary material for: Differential proteomic analysis of fetal and geriatric lumbar nucleus pulposus: immunoinflammation and age-related intervertebral disc degeneration
Source: BMC Musculoskelet Disord. 2020 Jun 2;21:339. doi: 10.1186/s12891-020-03329-8 (PMC7265631; doi:10.1186/s12891-020-03329-8)
Supplement: Supplementary file 1 — Additional file 1. Parameter Settings. Detailed parameter settings for the HPLC fraction sequence, LC-MS/MS analysis, database search, PRM analysis, Skyline analysis and database URLs. [file 12891_2020_3329_MOESM1_ESM.pdf]

HPLC Fraction Sequence

The fractions were as follows: final fraction 1= 1, 19, 37, and 55; final fraction 2= 2, 20, 38, and 56; final fraction 3= 3, 21, 39, and 57; final fraction 4= 4, 22, 40, and 58; final fraction 5= 5, 23, 41, and 59; final fraction 6= 6, 24, 42, and 60; final fraction 7= 7, 25, and 43; final fraction 8= 8, 26, and 44; final fraction 9= 9, 27, and 45; final fraction 10= 10, 28, and 46; final fraction 11= 11, 29, and 47; final fraction 12= 12, 30, and 48; final fraction 13= 13, 31, and 49; final fraction 14= 14, 32, and 50; final fraction 15= 15, 33, and 51; final fraction 16= 16, 34, and 52; and final fraction 17= 17, 35, and 53; final fraction 18= 18, 36, and 54.

LC-MS/MS Analysis Parameters

The parameter settings were as follows: the electrospray voltage applied was 2.0 kV, and the normalized collision energy (NCE) was 35. The scan range was 350 m/z to 1550 m/z for full MS, and the peptides were detected at a resolution of 60,000 in the Orbitrap. The scan range started at 100 m/z, and the resolution was set at 30,000 for MS/MS. A data-dependent acquisition (DDA) procedure was used to collect information, and an efficient scan mode was set at a maximum IT of 100 ms, an automatic gain control (AGC) of 5E4, a signal threshold of 5000 ions/s and a dynamic exclusion of 30 s.

Database Search Parameters

The parameter settings were as follows: a cleavage enzyme mode of trypsin/P, a maximum missed cleavage of 2, a minimum peptide length of 7, a maximum number of modifications of 5, a unique + razor score ≥ 2, a mass tolerance for precursor ions of 20 ppm in the first search and 5 ppm in the

main search, a mass tolerance for fragment ions of 0.02 Da, a fixed modification of alkylation on Cys, variable modifications acetylation on an N-terminal residue and oxidation on Met, a quantitative method of TMT-10 plex, and a false discovery rate (FDR) < 1%.

Software and Database URL

UniProt-GOA database: http://www.ebi.ac.uk/GOA/

InterProScan software: http://www.ebi.ac.uk/interpro/interproscan.html

InterPro database: http://www.ebi.ac.uk/interpro/

Kyoto Encyclopedia of Genes and Genomes (KEGG) database: https://www.genome.jp/

WoLF PSORT: https://www.genscript.com/psort/wolf_psort.html

PRM Analysis Parameters

The parameter settings were as follows: the electrospray voltage applied was 2.0 kV, and the NCE was 27. The scan range was 450 m/z to 1000 m/z for a full scan, and the resolution for Orbitrap detection was set to 70,000 detected in the Orbitrap. The fragments were selected for MS/MS and detected at a resolution of 17,500. The AGC was set at 3E6 for full MS and 1E5 for MS/MS. The maximum IT was set at 50 ms for full MS and 120 ms for MS/MS. The isolation window for MS/MS was set at 1.6 m/z.

Skyline Parameters

The parameter settings were as follows: a cleavage enzyme mode of trypsin [KR/P], a maximum missed cleavage of 0, a peptide length of 7 to 25, a fixed modification of carbamidomethyl on Cys,

precursor charges of 2 and 3, an ion charge of 1, and ion types of b and y. The product ions were set from ion 3 to the last ion, and the mass tolerance for fragment ions was set to 0.02 Da.
