# Supplementary material for: Differential proteomic analysis of fetal and geriatric lumbar nucleus pulposus: immunoinflammation and age-related intervertebral disc degeneration
Source: BMC Musculoskelet Disord. 2020 Jun 2;21:339. doi: 10.1186/s12891-020-03329-8 (PMC7265631; doi:10.1186/s12891-020-03329-8)
Supplement: Supplementary file 2 — Additional file 2. Quality control plots. The mass error and length distributions of peptides. [file 12891_2020_3329_MOESM2_ESM.pdf]

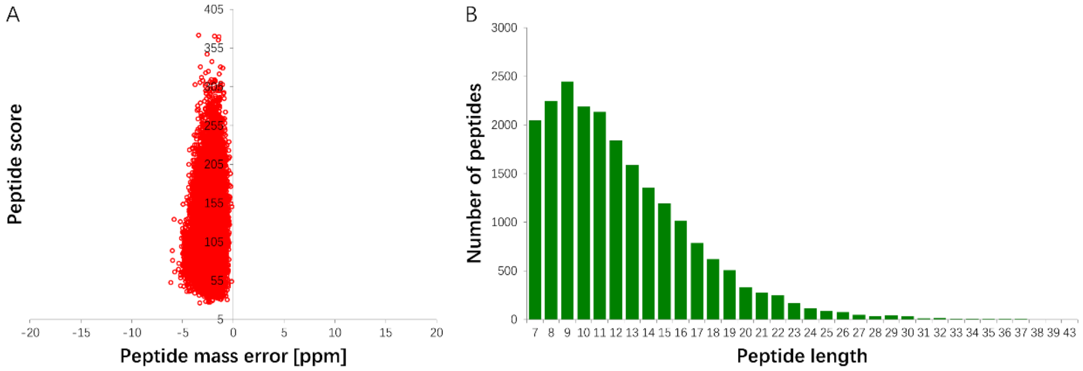

Figure. Peptide mass error and peptide length. (A) All the identified peptides were examined by mass error distribution. The mass errors were concentrated in the range below 5 ppm, indicating that they met the requirements. (B) The lengths of most peptides were between 8 and 20 amino acids, consistent with the typical lengths of trypsin-digested peptides and indicating that the sample preparation method met the standard.
