# Supplementary material for: Differential proteomic analysis of fetal and geriatric lumbar nucleus pulposus: immunoinflammation and age-related intervertebral disc degeneration
Source: BMC Musculoskelet Disord. 2020 Jun 2;21:339. doi: 10.1186/s12891-020-03329-8 (PMC7265631; doi:10.1186/s12891-020-03329-8)
Supplement: Supplementary file 4 — Additional file 4. GO annotation classification. [file 12891_2020_3329_MOESM4_ESM.docx]

Table 1. Upregulated GO annotation classification (Geriatric/Fetal).

| GO Terms Level 1 | GO Terms Level 2 | No. of Protein | GO Terms Level 1 | GO Terms Level 2 | No. of Protein |
| --- | --- | --- | --- | --- | --- |
| Biological Process | cellular process | 516 | Cellular Component | cell | 521 |
|  | single-organism process | 504 |  | organelle | 507 |
|  | biological regulation | 444 |  | extracellular region | 458 |
|  | metabolic process | 385 |  | membrane | 305 |
|  | response to stimulus | 380 |  | membrane-enclosed lumen | 203 |
|  | localization | 288 |  | macromolecular complex | 164 |
|  | multicellular | 267 |  | cell junction | 57 |
|  | organismal process |  |  | supramolecular complex | 28 |
|  | cellular component | 224 |  | synapse | 25 |
|  | organization or biogenesis |  | Molecular Function | binding | 549 |
|  | developmental process | 222 |  | catalytic activity | 243 |
|  | signaling | 200 |  | molecular function regulator | 90 |
|  | immune system process | 189 |  | structural molecule activity | 43 |
|  | multi-organism process | 105 |  | transporter activity | 41 |
|  | locomotion | 80 |  | signal transducer activity | 33 |
|  | biological adhesion | 66 |  | molecular transducer activity | 30 |
|  | reproduction | 40 |  | antioxidant activity | 15 |

Table 2. Downregulated GO annotation classification (Geriatric/Fetal).

| GO Terms Level 1 | GO Terms Level 2 | No. of Protein | GO Terms Level 1 | GO Terms Level 2 | No. of Protein |
| --- | --- | --- | --- | --- | --- |
| Biological Process | cellular process | 668 | Cellular Component | extracellular region | 337 |
|  | single-organism process | 538 |  | membrane-enclosed lumen | 335 |
|  | biological regulation | 487 |  | macromolecular complex | 328 |
|  | metabolic process | 486 |  | cell junction | 84 |
|  | response to stimulus | 338 |  | supramolecular complex | 53 |
|  | cellular component | 323 |  | synapse | 39 |
|  | organization or biogenesis |  | Molecular Function | binding | 690 |
|  | localization | 322 |  | catalytic activity | 288 |
|  | multicellular | 242 |  | structural molecule activity | 84 |
|  | organismal process |  |  | molecular function regulator | 60 |
|  | developmental process | 224 |  | transporter activity | 36 |
|  | signaling | 185 |  | signal transducer activity | 20 |
|  | multi-organism process | 136 |  | transcription factor | 20 |
|  | immune system process | 117 |  | activity, protein binding |  |
| Cellular Component | cell | 713 |  | nucleic acid binding | 19 |
|  | organelle | 668 |  | transcription factor activity |  |
|  | membrane | 373 |  |  |  |
