# Supplementary material for: Differential proteomic analysis of fetal and geriatric lumbar nucleus pulposus: immunoinflammation and age-related intervertebral disc degeneration
Source: BMC Musculoskelet Disord. 2020 Jun 2;21:339. doi: 10.1186/s12891-020-03329-8 (PMC7265631; doi:10.1186/s12891-020-03329-8)
Supplement: Supplementary file 5 — Additional file 5. PRM fragment ion peak maps. [file 12891_2020_3329_MOESM5_ESM.pdf]

Fragment Ion Peak Maps

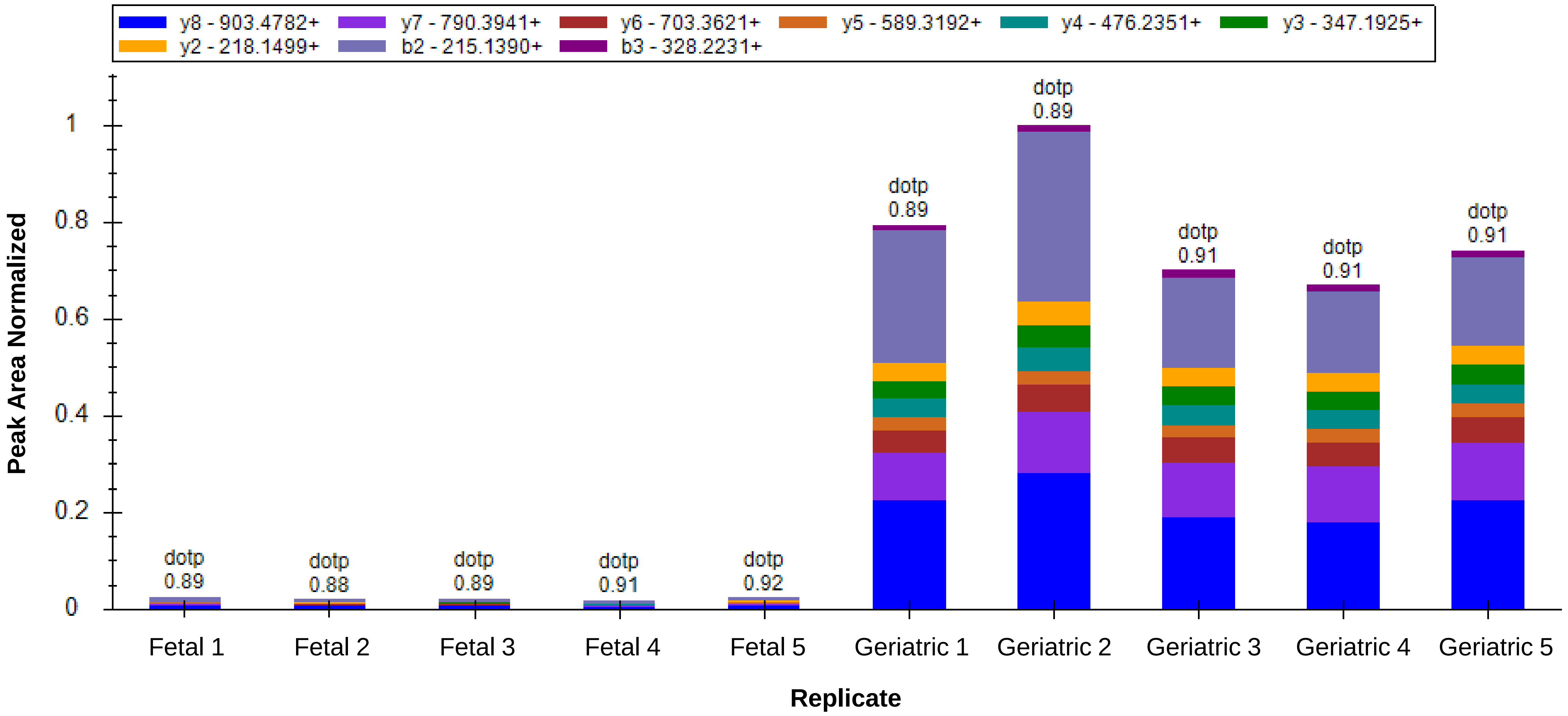

Protein Name: Clusterin (P10909), Unique Peptide Used: TLLSNLEEAK, Retention Time: 22.61

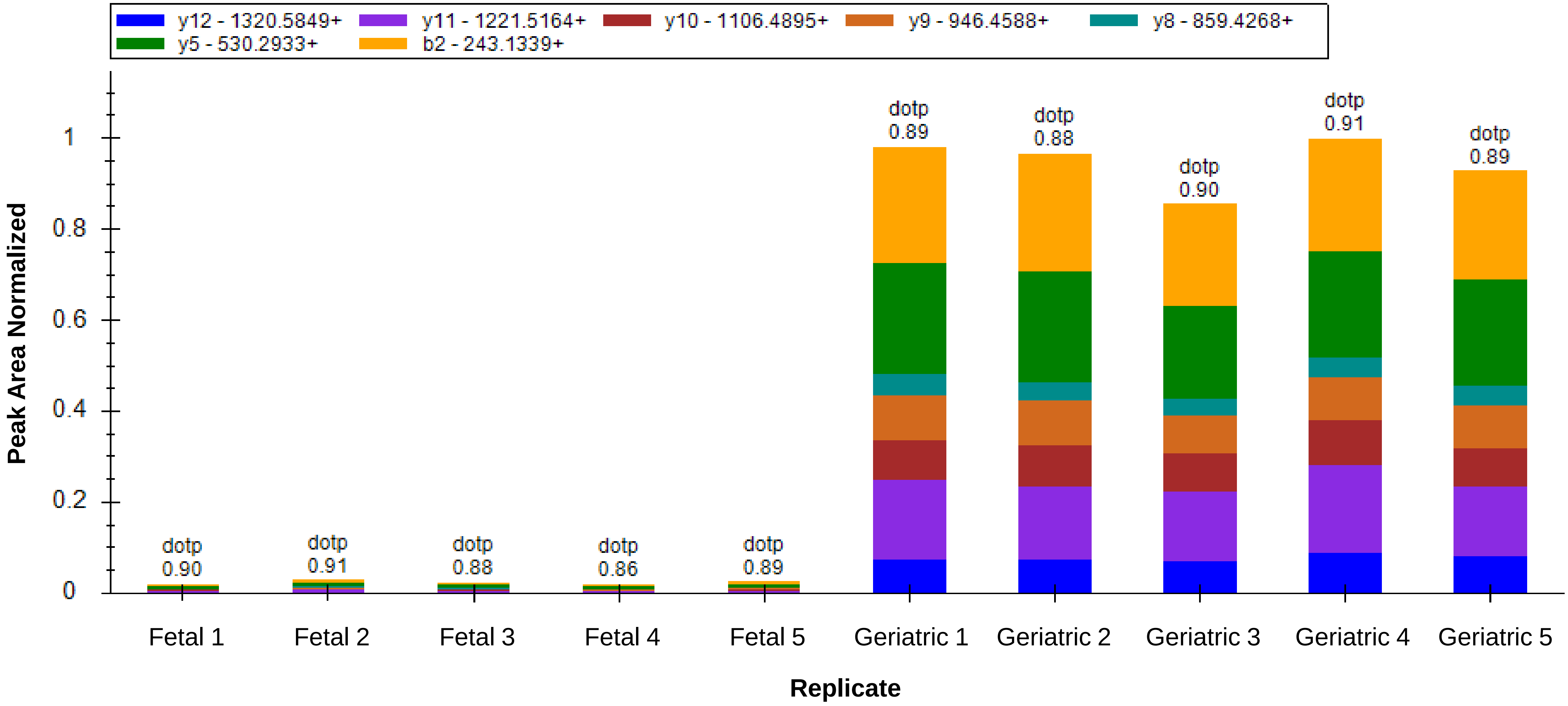

Protein Name: Clusterin (P10909), Unique Peptide Used: EILSVDCSTNNPSQAK, Retention Time: 12.92

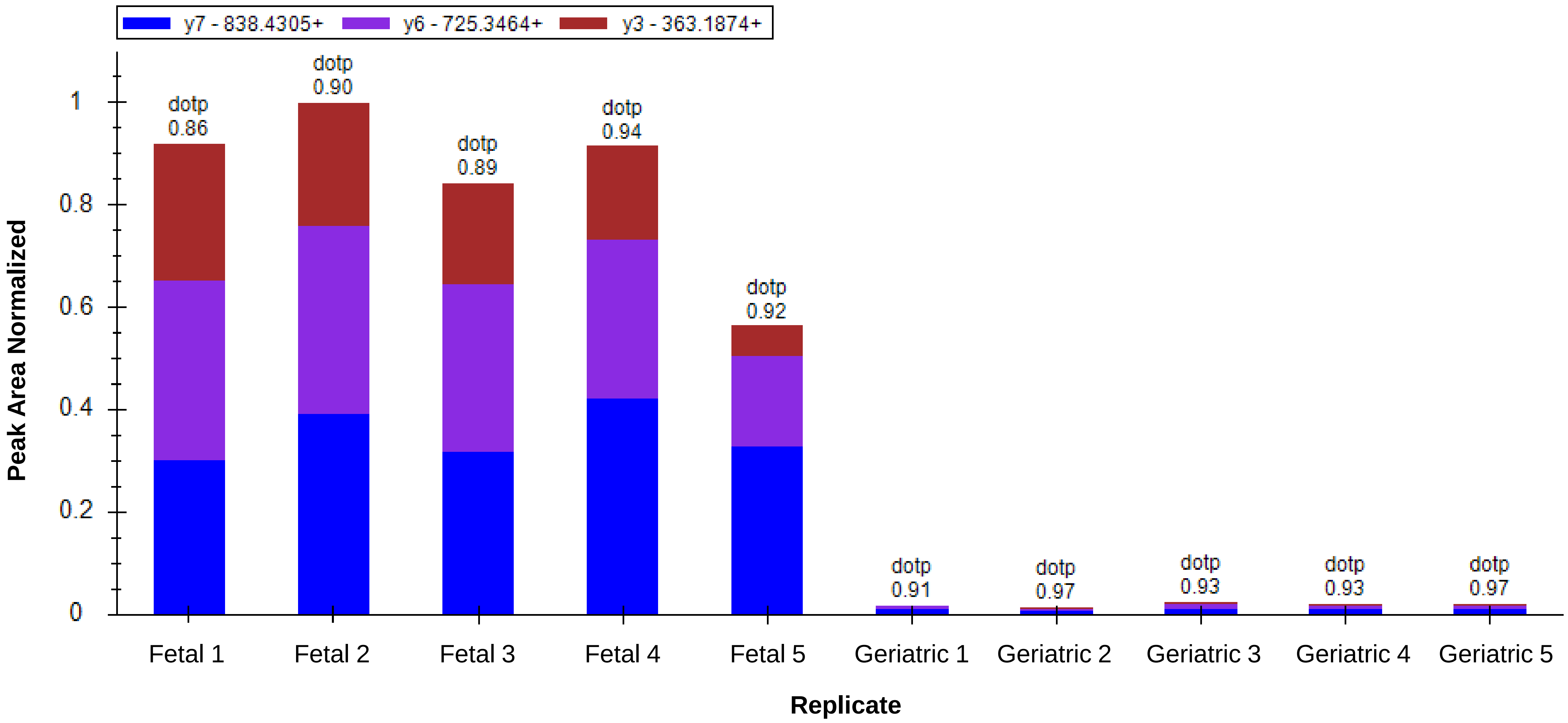

Protein Name: Matrilin-3 (O15232), Unique Peptide Used: IEFQLQAYTDK, Retention Time: 28.37

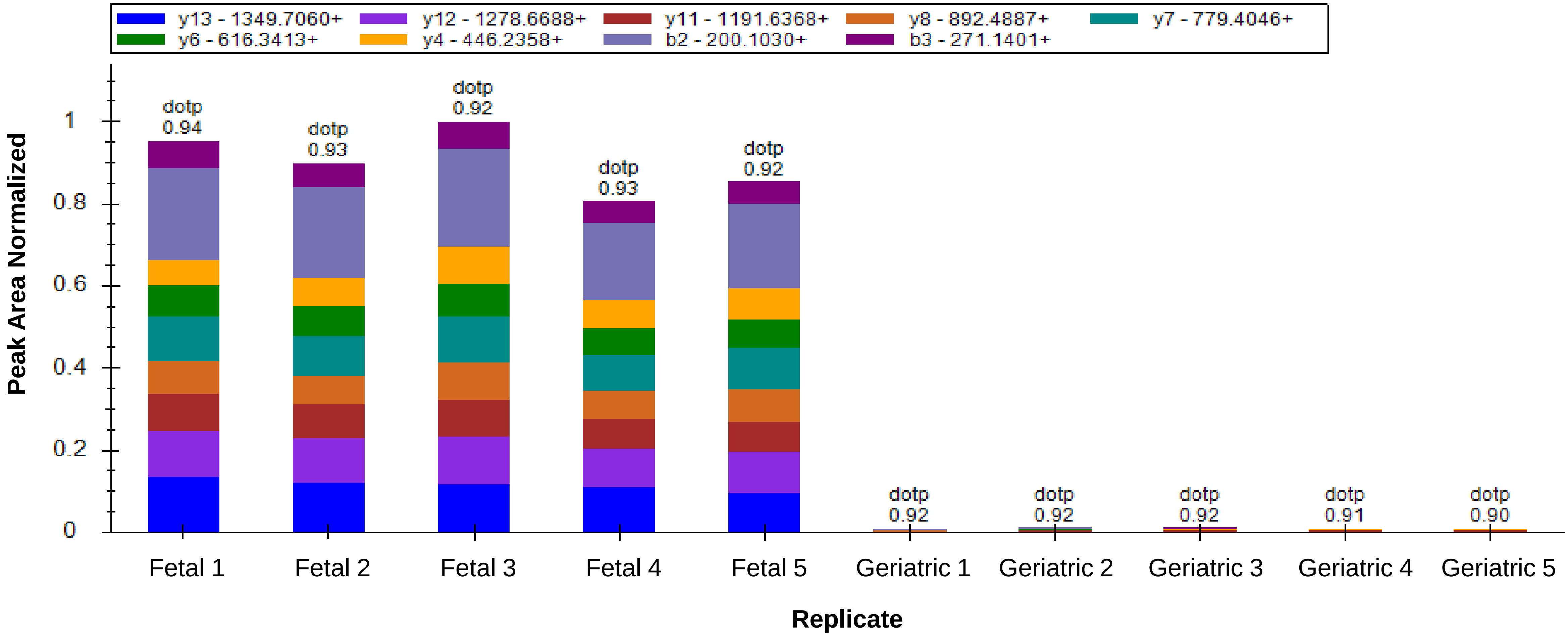

Protein Name: Matrilin-3 (O15232), Unique Peptide Used: AQASGIELYAVGVDR, Retention Time: 27.89

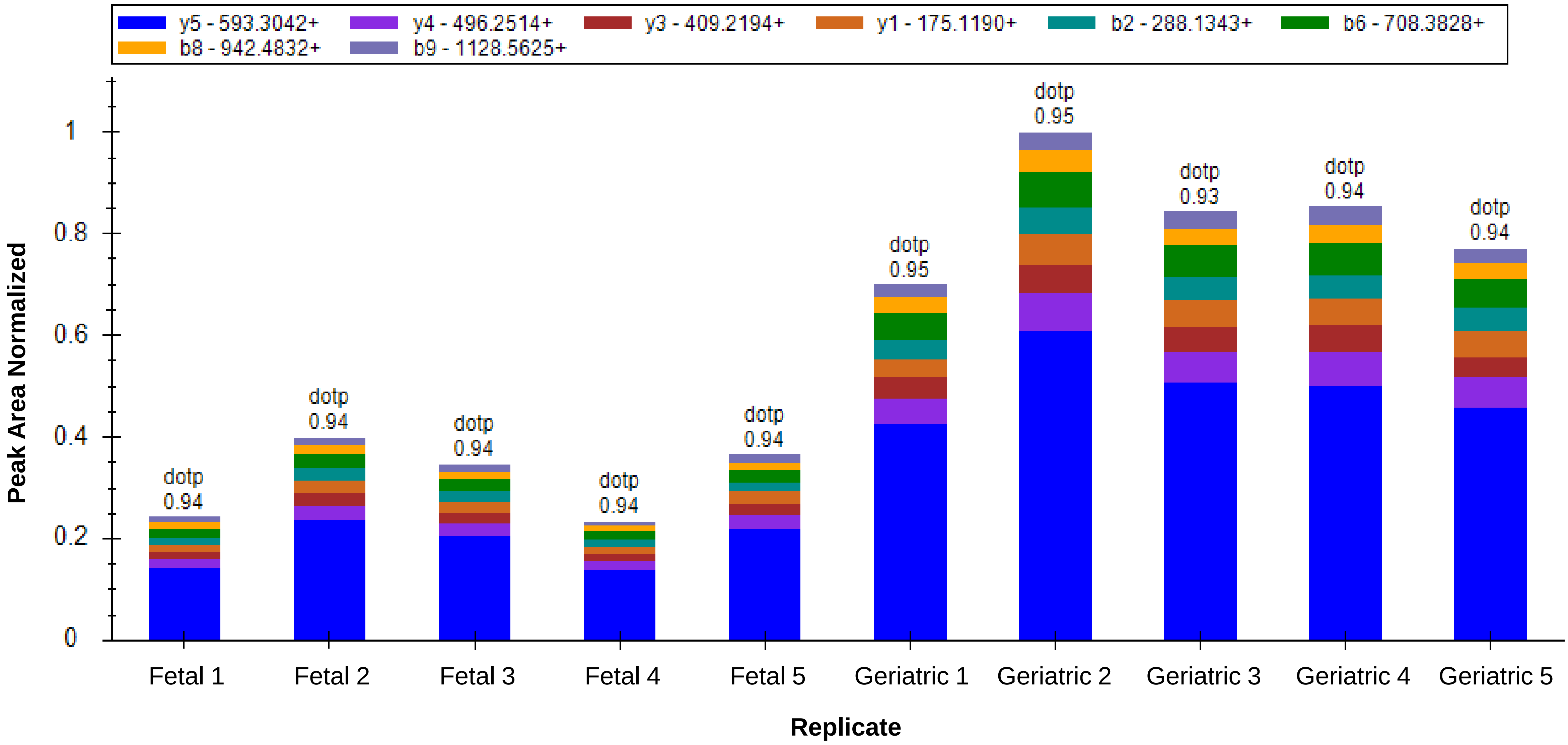

Protein Name: Lactadherin (Q08431), Unique Peptide Used: TWGLHLFSWNPSYAR, Retention Time: 43.74

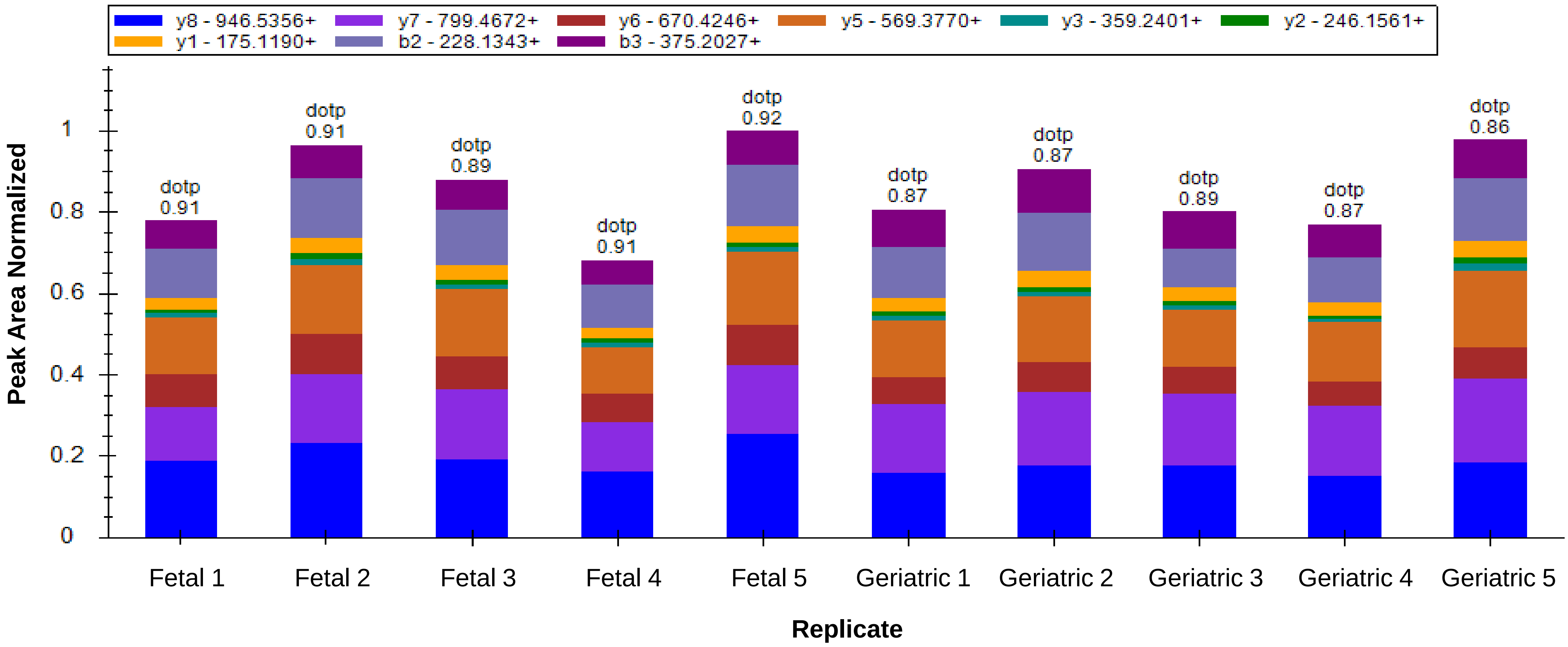

Protein Name: Lactadherin (Q08431), Unique Peptide Used: NLFETPILAR, Retention Time: 34.05

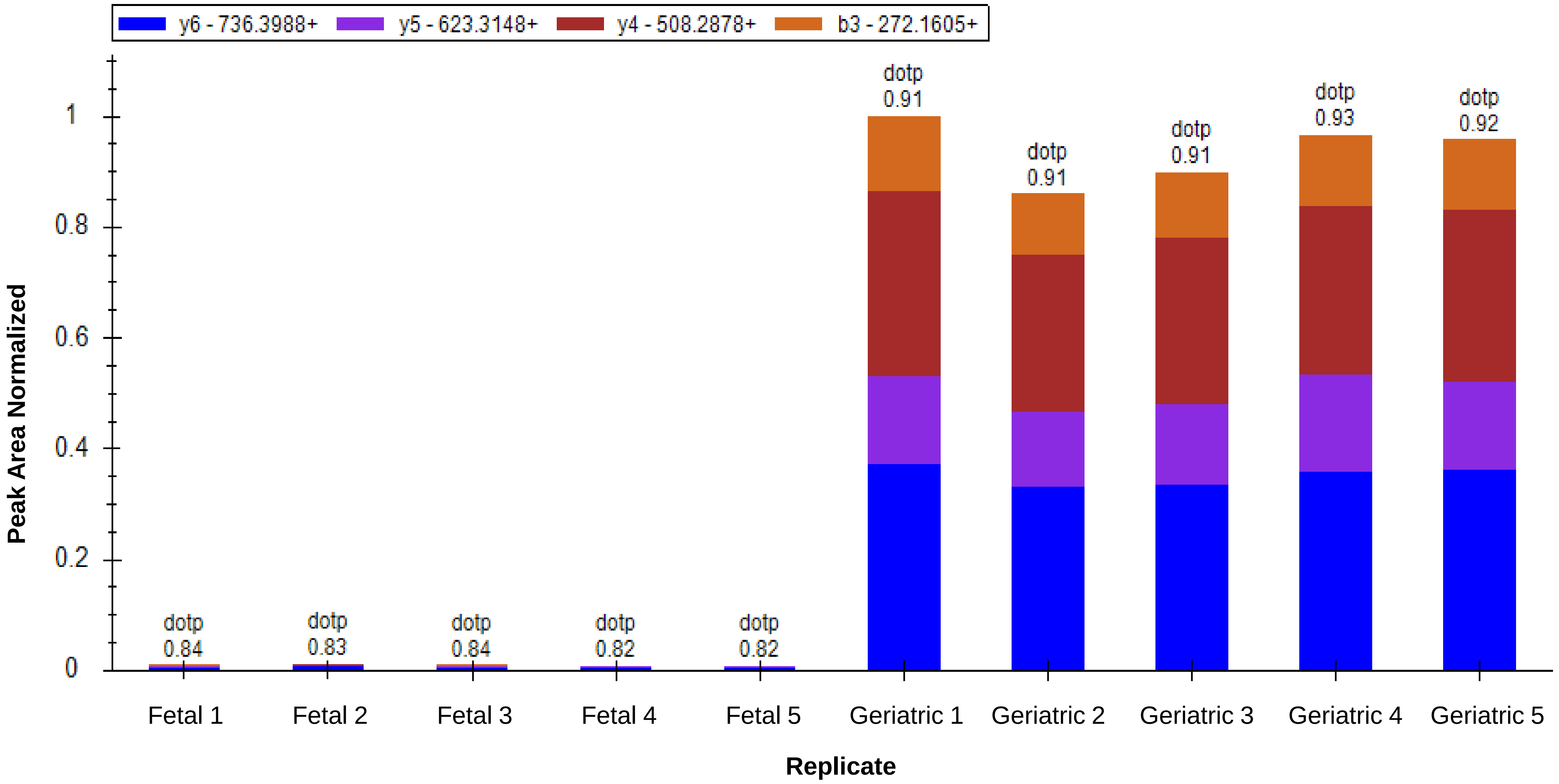

Protein Name: Tumor necrosis factor-inducible gene 6 protein (P98066),
Unique Peptide Used: TGIIDYGIR, Retention Time: 20.67

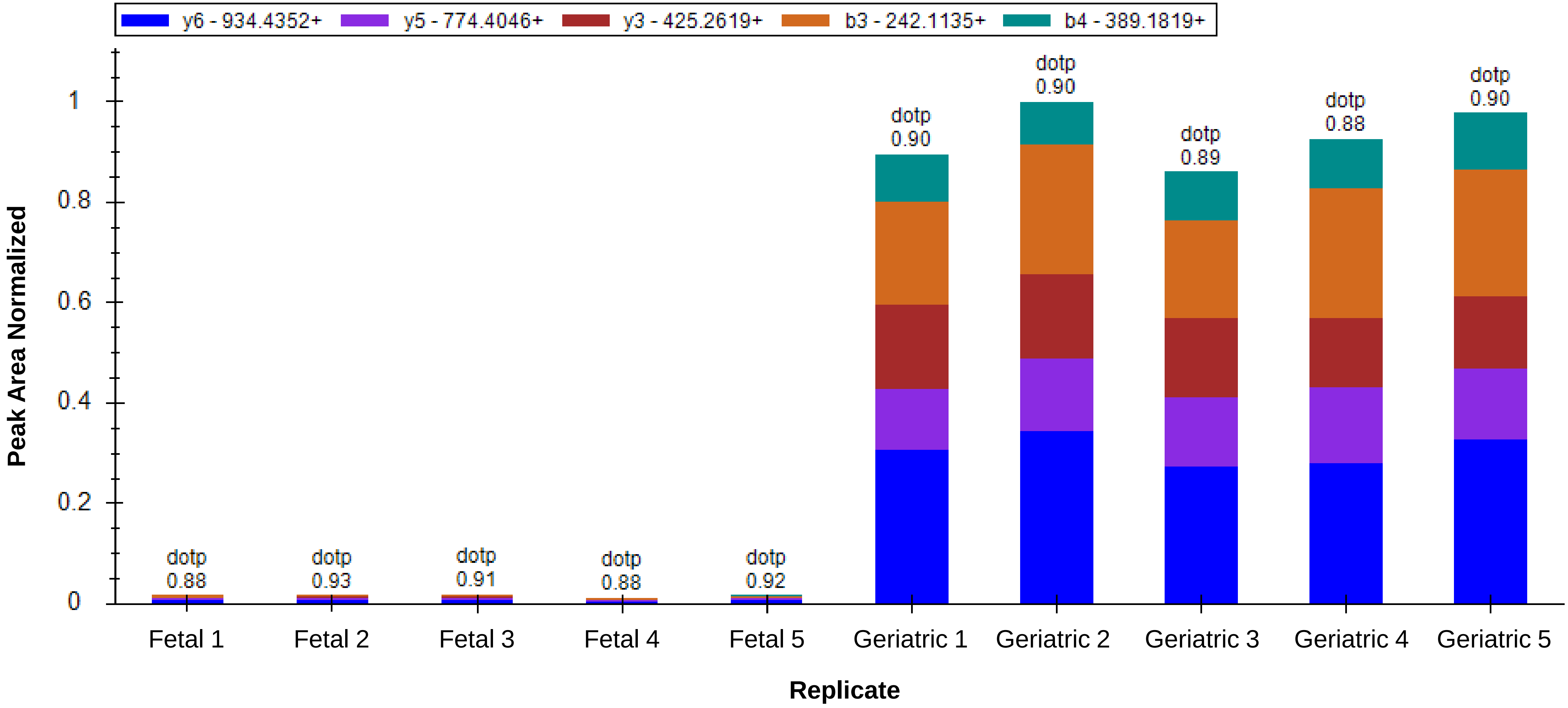

Protein Name: Tumor necrosis factor-inducible gene 6 protein (P98066),
Unique Peptide Used: SPGFPNEYEDNQICYWHIR, Retention Time: 34.57

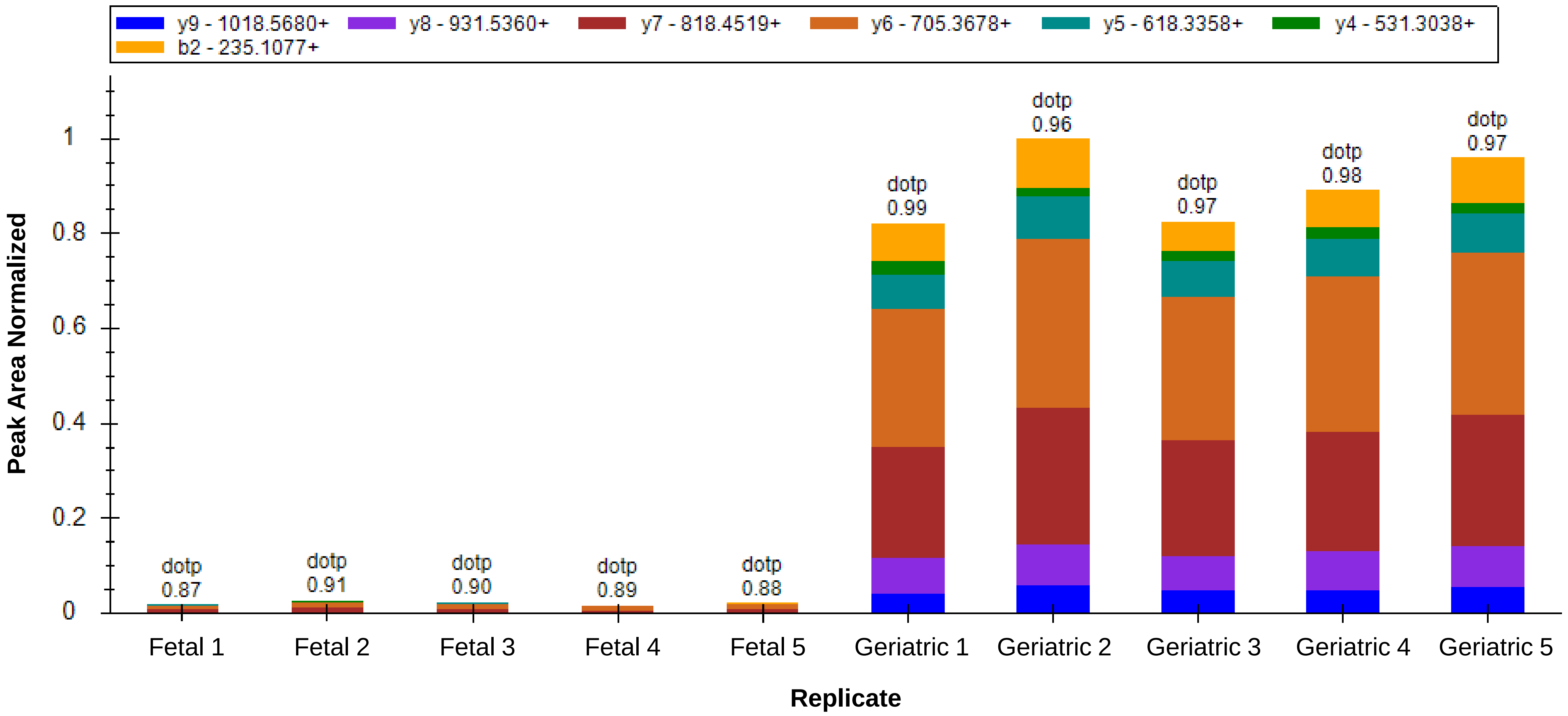

Protein Name: Matrix-remodeling-associated protein 5 (Q9NR99),
Unique Peptide Used: FSILSSGWLR, Retention Time: 41.78

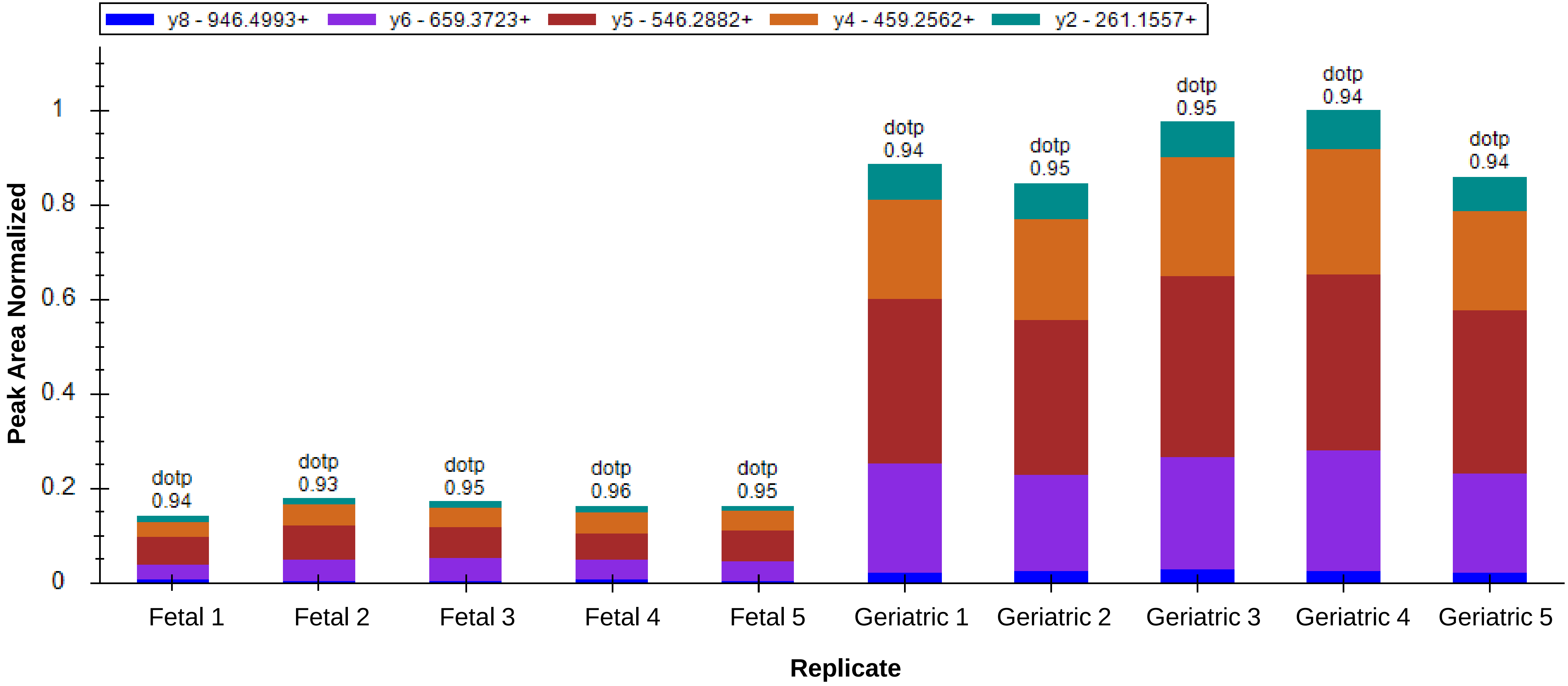

Protein Name: Matrix-remodeling-associated protein 5 (Q9NR99),
Unique Peptide Used: VTWLSPTNK, Retention Time: 15.44

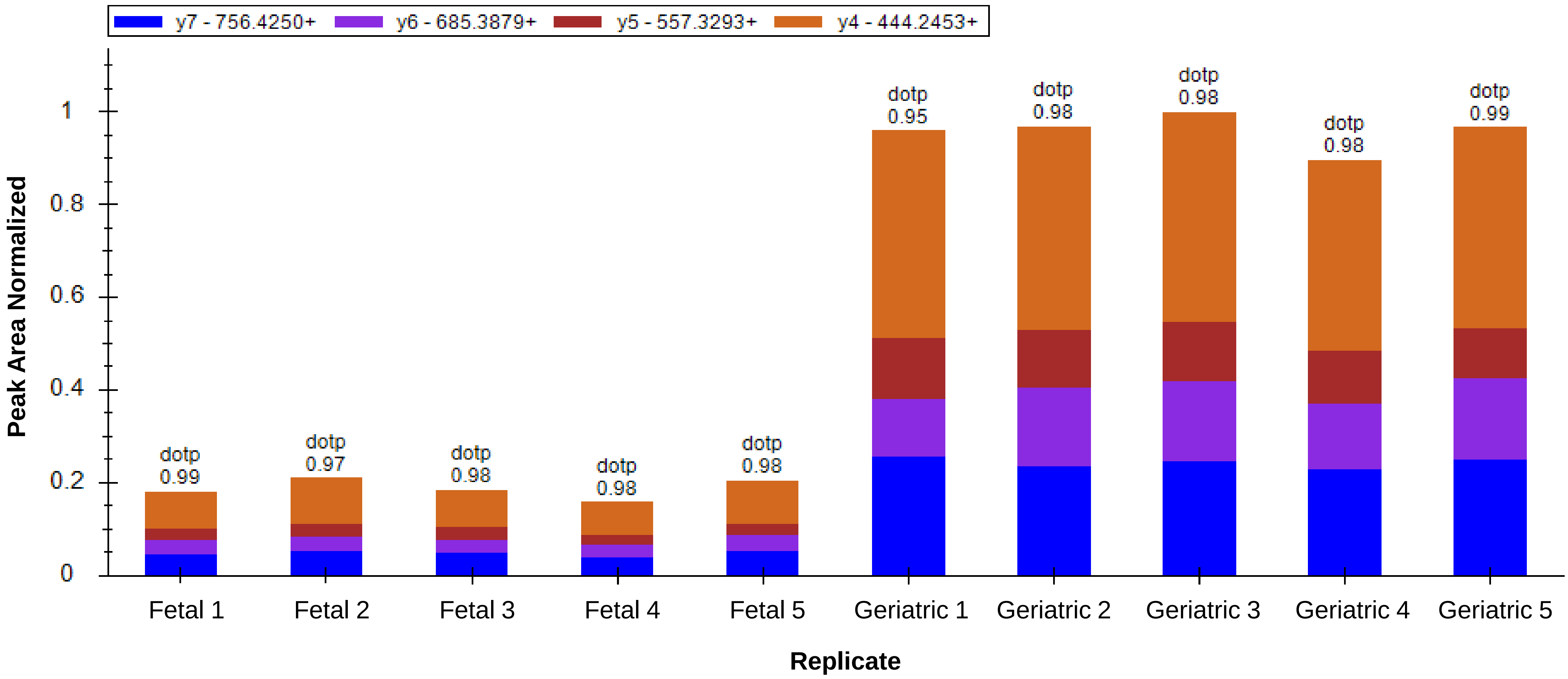

Protein Name: Extracellular matrix protein 1 (Q16610),
Unique Peptide Used: LLPAQLPAEK, Retention Time: 17.51

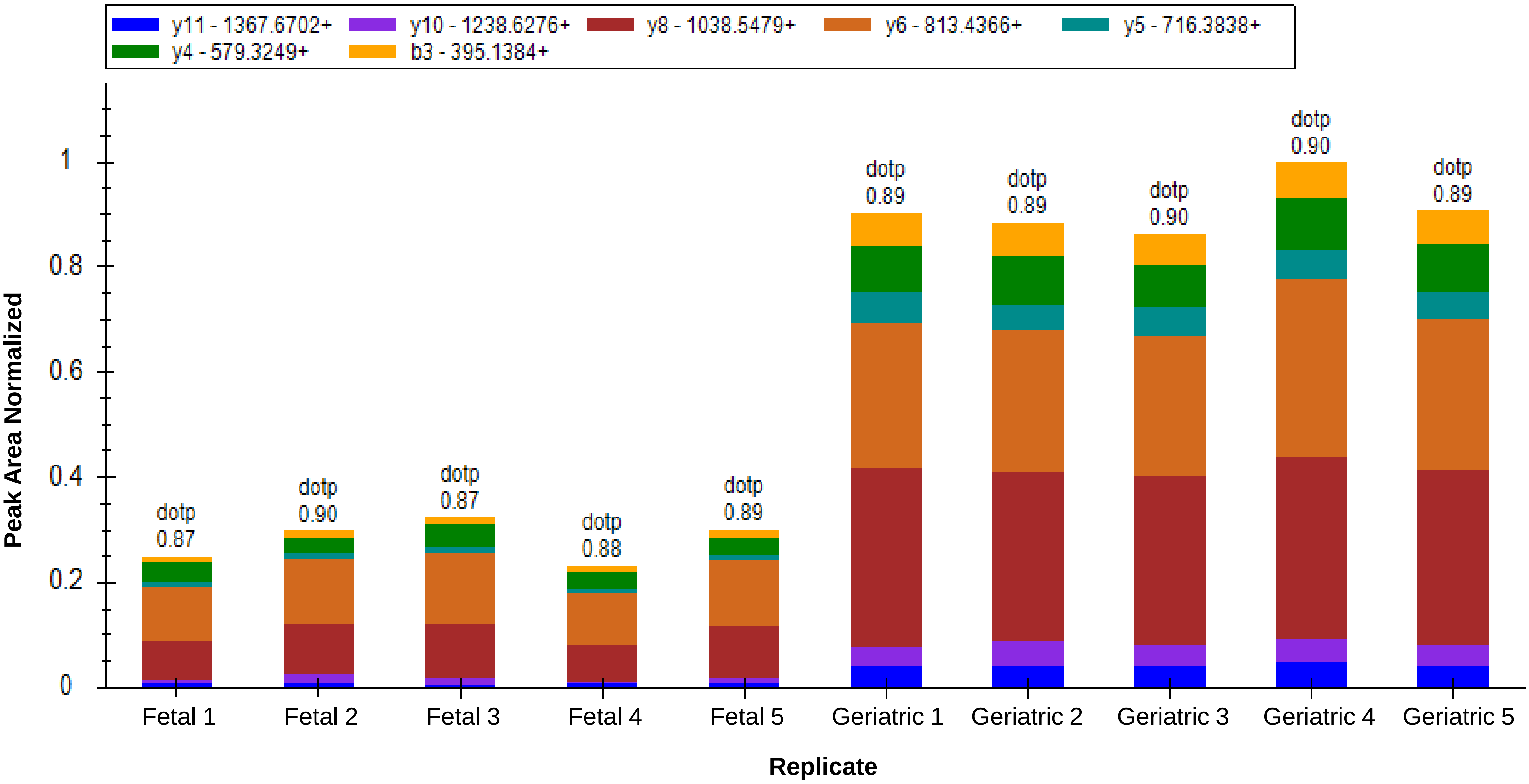

Protein Name: Extracellular matrix protein 1 (Q16610),
Unique Peptide Used: FSCFQEEAPQPHYQLR, Retention Time: 21.45

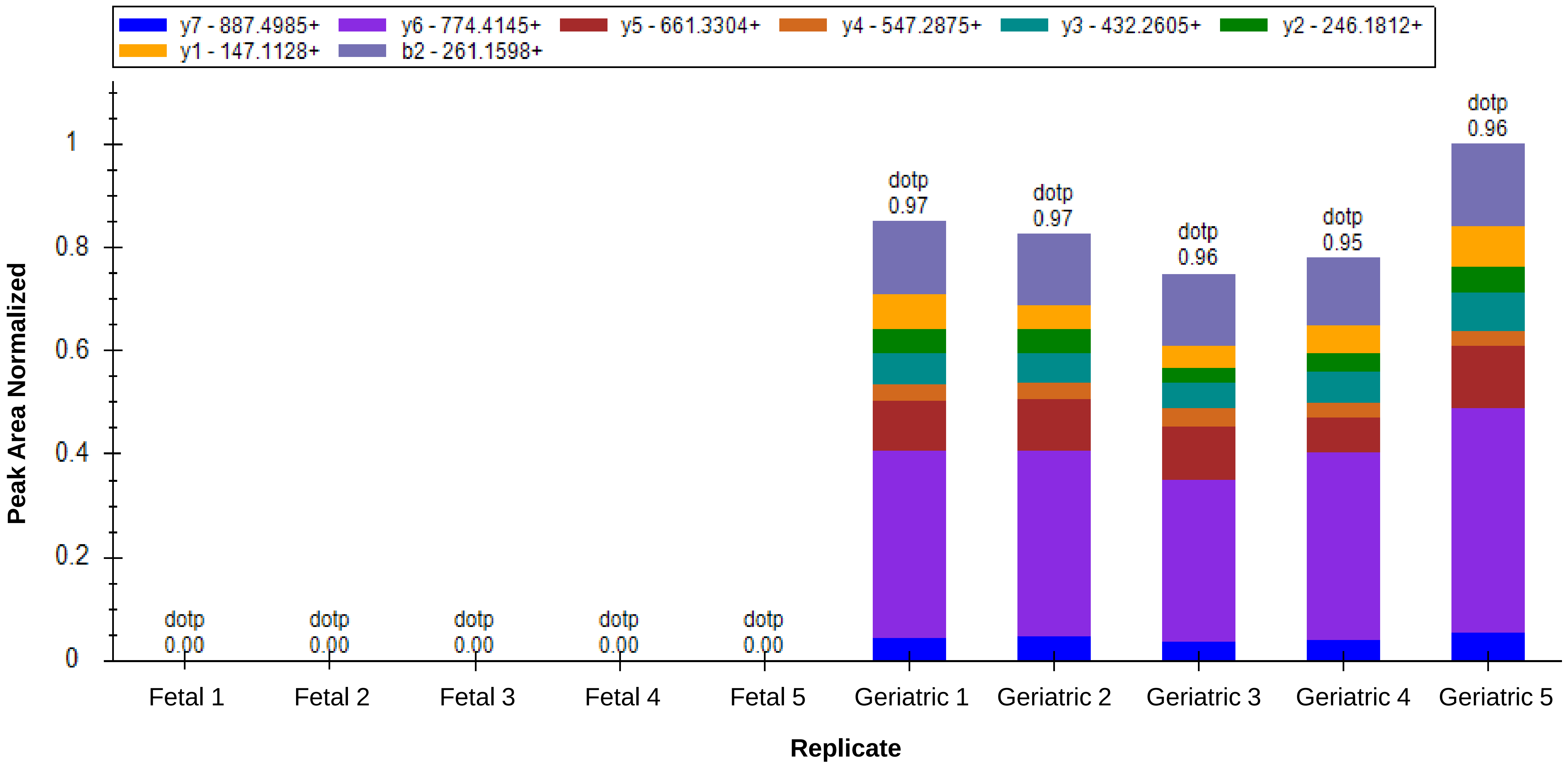

Protein Name: Plasminogen activator inhibitor 1 (P05121),
Unique Peptide Used: FIINDWVK, Retention Time: 33.53

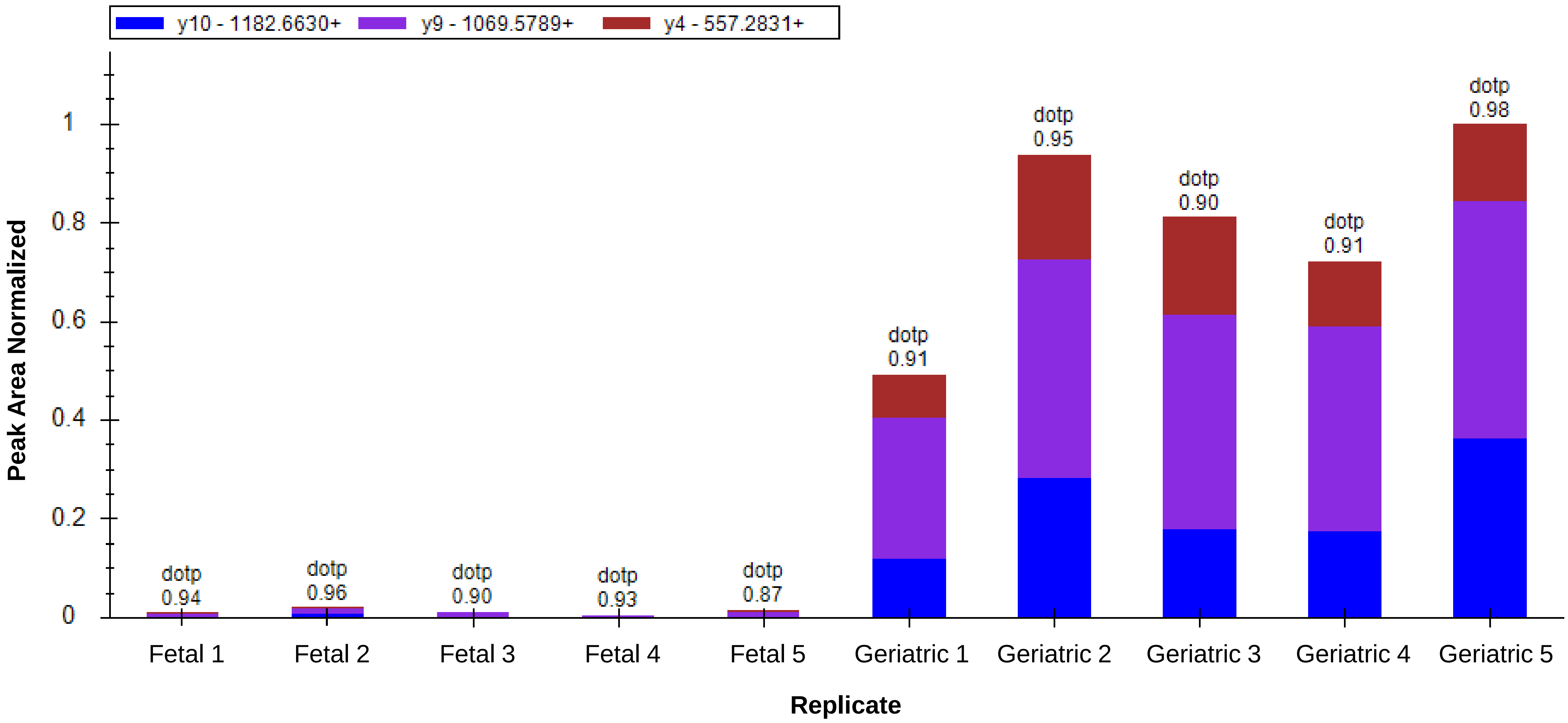

Protein Name: Plasminogen activator inhibitor 1 (P05121),
Unique Peptide Used: EVPLSALTNILSAQLISHWK, Retention Time: 55.78

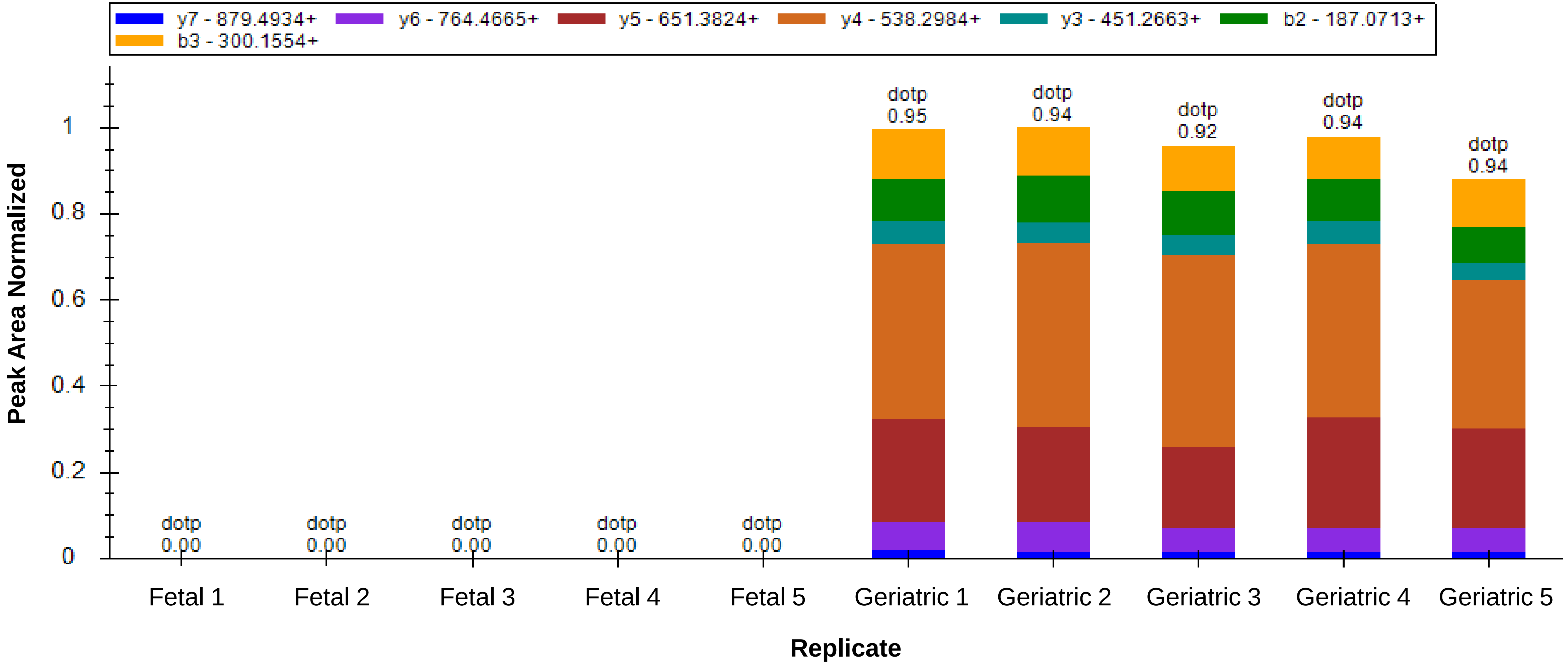

Protein Name: Interleukin-11 (P20809),
Unique Peptide Used: ADLLSYLR, Retention Time: 34.4

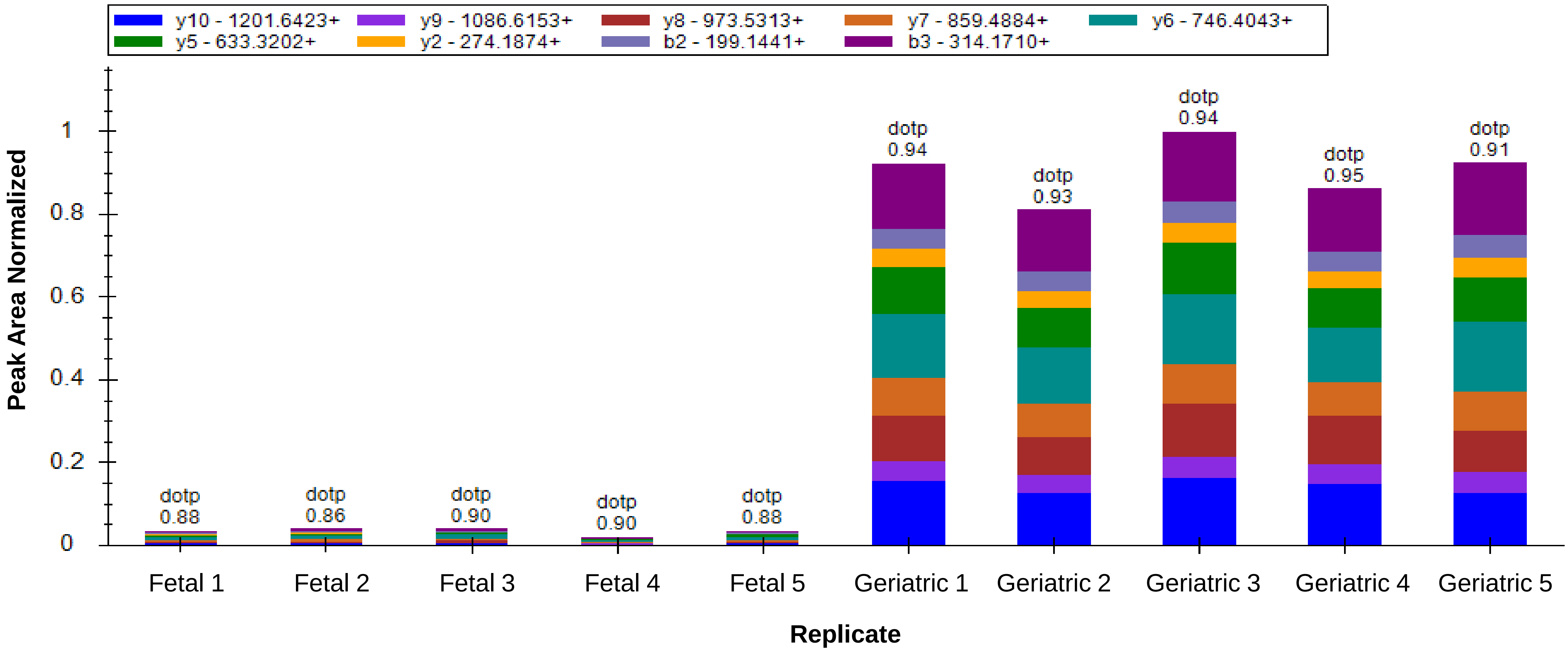

Protein Name: Platelet-derived growth factor C (Q9NRA1),
Unique Peptide Used: VVDLNLLTEEVR, Retention Time: 38.38

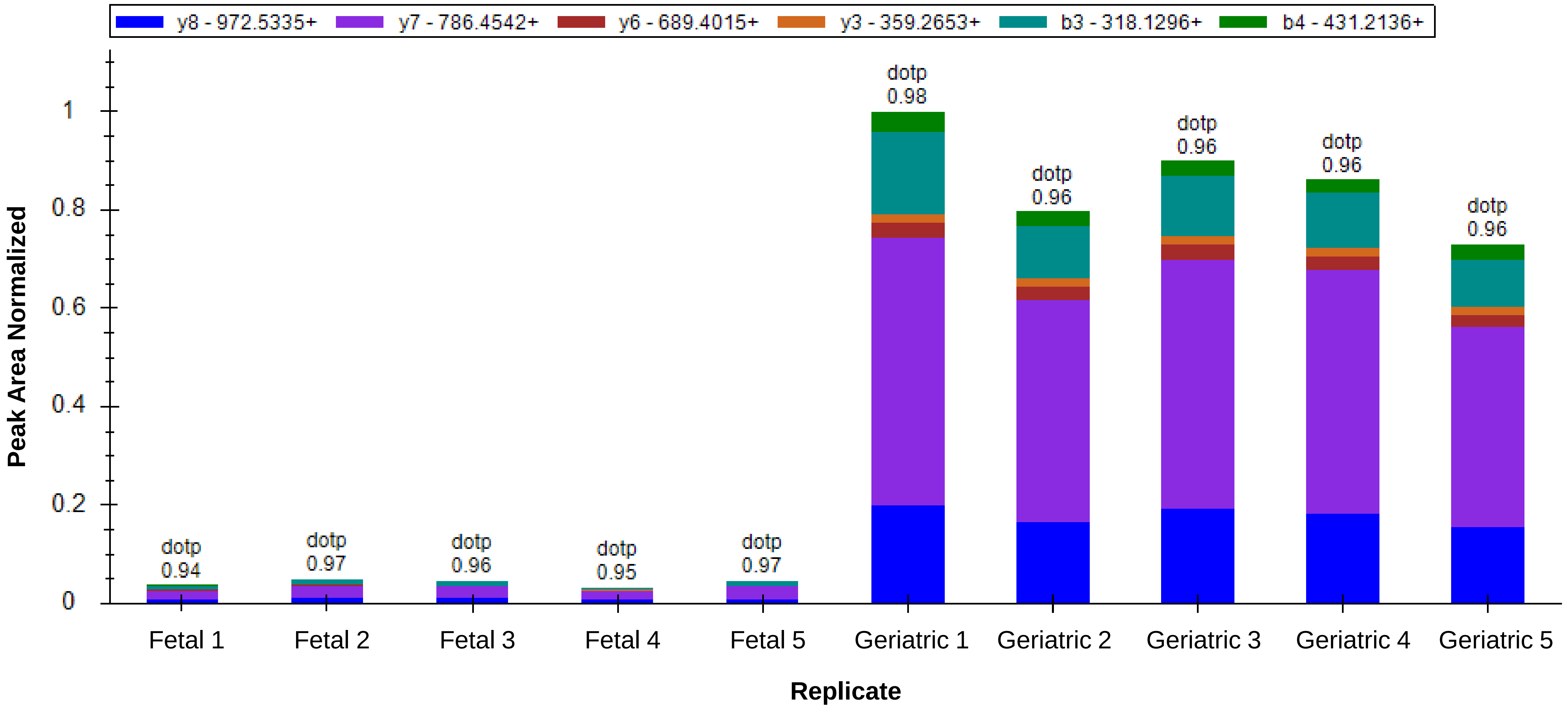

Protein Name: Platelet-derived growth factor C (Q9NRA1),
Unique Peptide Used: TDTIFWPGCLLVK, Retention Time: 48.72

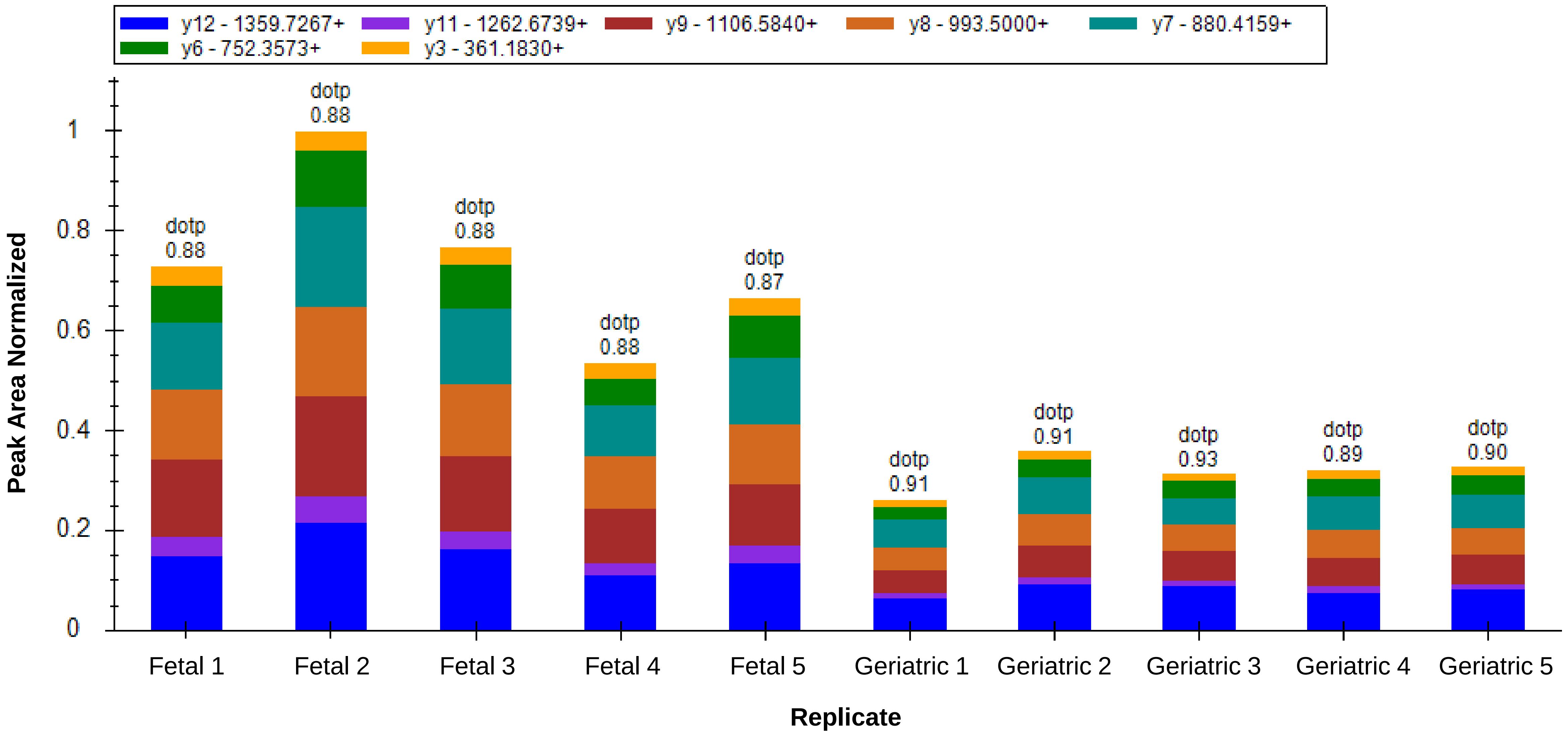

Protein Name: Heat shock cognate 71 kDa protein (P11142),
Unique Peptide Used: QTQTFTTYSDNQPGVLIQVYEGER, Retention Time: 39.3

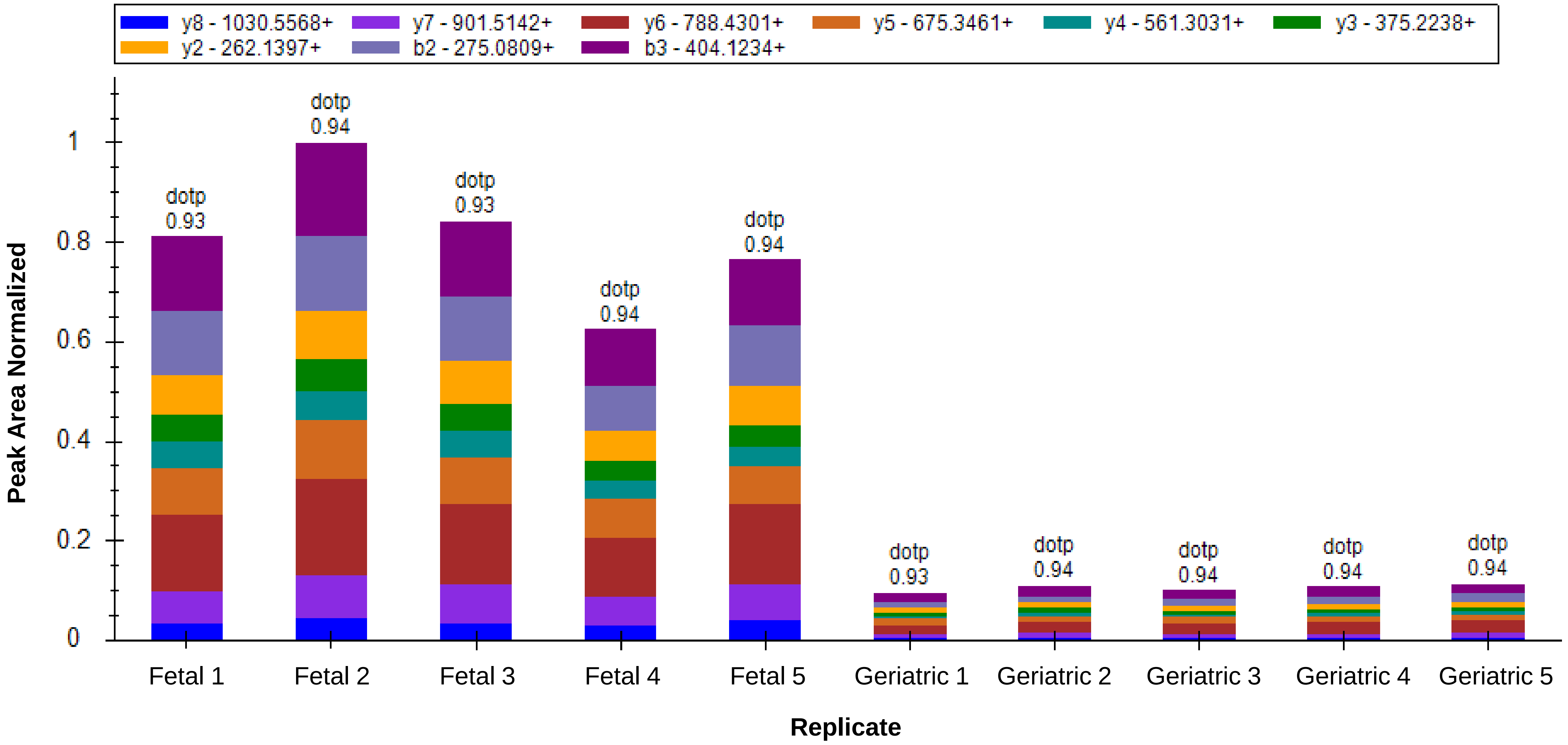

Protein Name: Heat shock cognate 71 kDa protein (P11142),
Unique Peptide Used: CNEIINWLDK, Retention Time: 34.67

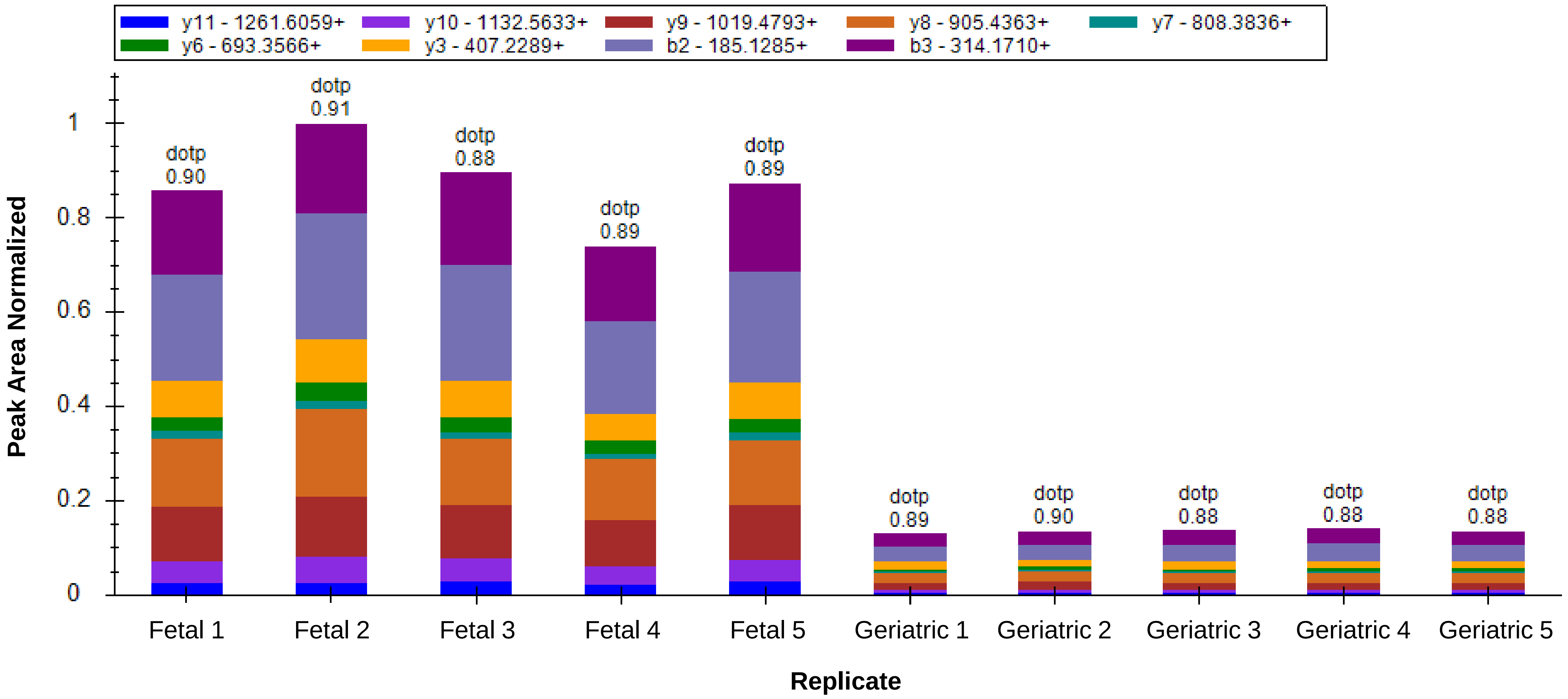

Protein Name: Hsc70-interacting protein (P50502),
Unique Peptide Used: AIEINPDSAQPYK, Retention Time: 17.32

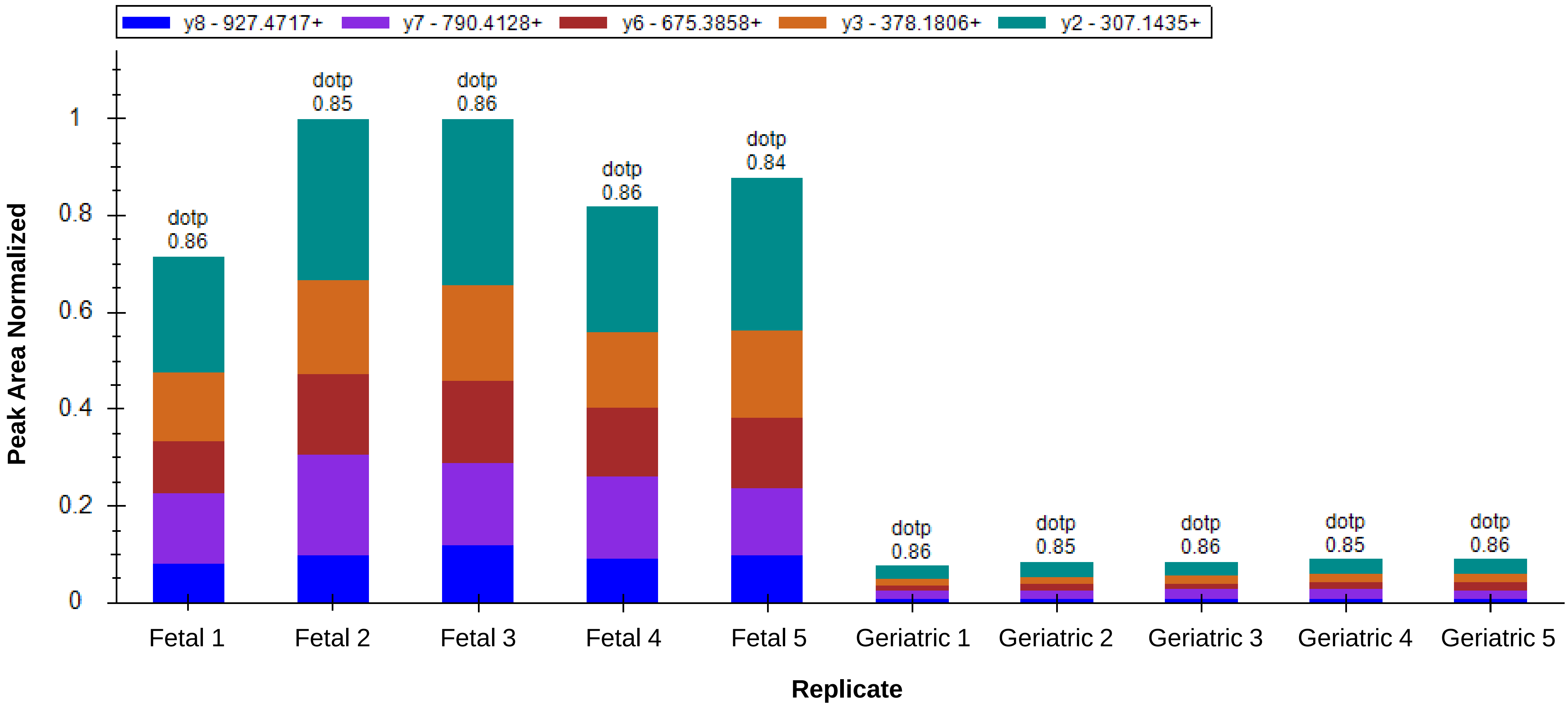

Protein Name: Hsc70-interacting protein (P50502),
Unique Peptide Used: LLGHWEEAAHDLALACK, Retention Time: 31.22
